# Breast tissue regeneration is driven by cell-matrix interactions coordinating multi-lineage stem cell differentiation through DDR1

**DOI:** 10.1038/s41467-021-27401-6

**Published:** 2021-12-10

**Authors:** Gat Rauner, Dexter X. Jin, Daniel H. Miller, Todd M. Gierahn, Carman M. Li, Ethan S. Sokol, Yu-Xiong Feng, Robert A. Mathis, J. Christopher Love, Piyush B. Gupta, Charlotte Kuperwasser

**Affiliations:** 1 Department of Developmental, Chemical & Molecular Biology, Tufts University, Boston, MA 02111 USA; 2 Whitehead Institute for Biomedical Research, Cambridge, MA 02142 USA; 3 Department of Biology, Massachusetts Institute of Technology, Cambridge, MA 02139 USA; 4 Koch Institute for Integrative Cancer Research, Massachusetts Institute of Technology, Cambridge, MA 02142 USA; 5 Department of Cell Biology, Ludwig Center at Harvard, Harvard Medical School, Boston, MA 02115 USA; 6 Broad Institute of MIT and Harvard, Cambridge, MA 02142 USA; 7 Ragon Institute of MGH, MIT and Harvard, Cambridge, MA 02129 USA; 8 Laboratory for the Convergence of Biomedical, Physical, and Engineering Sciences, Tufts University School of Medicine, Boston, MA 02111 USA

**Keywords:** Regeneration, Extracellular signalling molecules, Mammary stem cells, Biomedical engineering, Biomaterials - cells

## Abstract

Mammary morphogenesis is an orchestrated process involving differentiation, proliferation and organization of cells to form a bi-layered epithelial network of ducts and lobules embedded in stromal tissue. We have engineered a 3D biomimetic human breast that makes it possible to study how stem cell fate decisions translate to tissue-level structure and function. Using this advancement, we describe the mechanism by which breast epithelial cells build a complex three-dimensional, multi-lineage tissue by signaling through a collagen receptor. Discoidin domain receptor tyrosine kinase 1 induces stem cells to differentiate into basal cells, which in turn stimulate luminal progenitor cells via Notch signaling to differentiate and form lobules. These findings demonstrate how human breast tissue regeneration is triggered by transmission of signals from the extracellular matrix through an epithelial bilayer to coordinate structural changes that lead to formation of a complex ductal-lobular network.

## Abstract (web summary)

Mammary morphogenesis is a complex process. Here the authors describe how stem cells build a three-dimensional self-organizing multi-lineage tissue by showing that positional signals from the extracellular matrix through the collagen receptor DDR1 lead stem cells to differentiate into multi-lineage committed multi-layered progeny.

## Introduction

Adult stem cells have the remarkable ability to generate architecturally complex tissues composed of multiple cell types. In the mammary gland, stem cells form a hollow epithelial tree composed of branched ducts that terminate in grape-like clusters called terminal ductal lobular units (TDLU). This tree has two layers: an inner layer of luminal cells that secrete milk into the internal space, and an outer basal layer of myoepithelial cells anchored to the surrounding matrix that contract to expel milk. Rare stem cells are peppered between these layers and give rise to luminal and myoepithelial cells by differentiating into unipotent progenitors that are committed to either the luminal or basal lineage.

It has long been recognized that mammary epithelial cells (MECs) have the capacity to self-organize in a 3D matrix, and that the extracellular matrix (ECM) provides essential signaling components that lead stem and progenitor cells to initiate the self-assembly and expansion of tissues ex-vivo^1,2^. It has also become clear that architecturally different tissues are optimally supported by distinctly different ECM compositions^3–5^. We recently described the establishment of a biomimetic 3D hydrogel scaffold in which human breast epithelial cells self-organize and morph into complex bi-layered ductal-lobular-alveolar structures that closely parallel their in-vivo counterparts^6,7^. This biomimetic ECM matches the physical, cellular, and microenvironmental properties of the human breast and supports long-term growth of an epithelial network while retaining stem cell hierarchy, differentiation, and hormonal responses^6,7^. The use of defined biomimetic scaffolds provides the opportunity to identify the signaling cues that direct tissue regeneration.

In this study, we venture to use biomimetic engineering to study mammary stem cell fate decisions and breast tissue regeneration. By screening for factors necessary for mammary stem cell (MaSC) self-renewal in 3D, we discover that the ECM transmits positional signals through the discoidin domain receptor tyrosine kinase 1 (DDR1), via Notch1 and its ligand Jagged-1. This cue stimulates coordinated differentiation of stem and progenitor cells into mature basal and luminal cells, thereby driving lobular-alveolar growth. Our findings demonstrate how the human breast epithelium regenerates itself by coupling stem cell fate decisions with spatial information from the ECM.

## Results

### Identification of DDR1 as a regulator of tissue regeneration

**Fig. 1:**
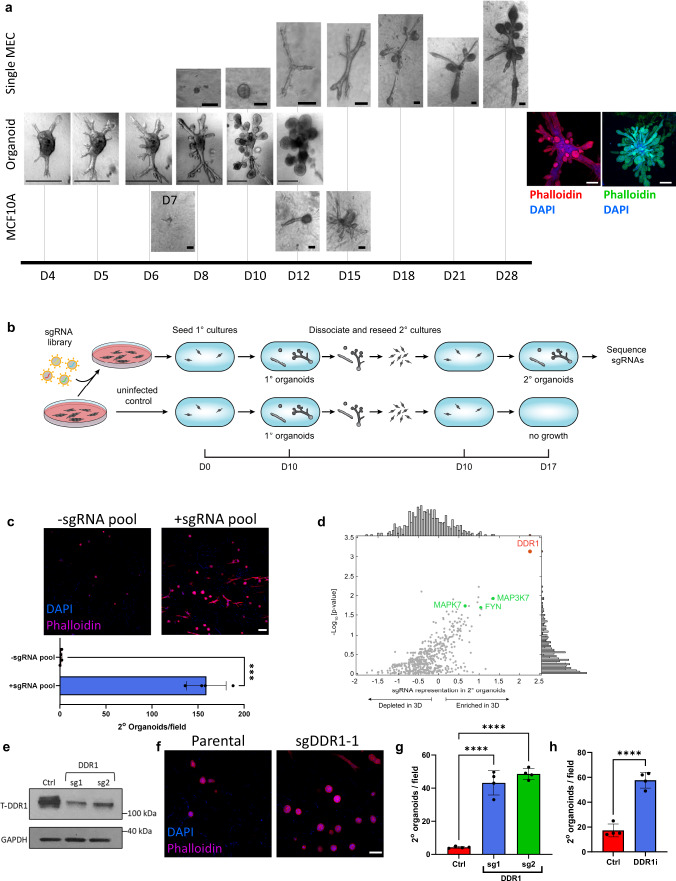
CRISPR screen in 3D mammary epithelial cell culture for regulators of stem cell self-renewal. **a** Development of primary breast cells (single MECs), primary organoids (Organoid MECs), and MCF10A cells in 3D hydrogels. Phalloidin-stained Type I and II lobules that developed from organoids are shown. Results are representative of at least 3 independent experiments. Scale bars = 100 µm (Single MECs and MCF10A), 200 µm (Phalloidin stained lobules) or 500 µm (Organoid MECs). **b** Schematic of pooled CRISPR screening strategy. **c** Phalloidin-stained secondary organoids from MCF10A cells with or without sgRNAs (top), and quantification of organoids (bottom). *P* = 0.0007 (two-sided Student’s *t*-test). Data were derived from *n* = 4 independently analyzed gels. Scale bar = 100 µm. **d** Screened kinases scored by significance relative to a null distribution using RIGER (*y*-axis) and by comparing the mean differential abundance of sgRNAs targeting the kinase (*x*-axis). Adjacent histograms indicate *p*-value distribution (right *y*-axis) and mean differential (top *x*-axis). RIGER’s *p*-values are adjusted for multiple tests. Significant genes previously implicated in cellular differentiation indicated in green. **e** Western blot for total DDR1 in parental MCF10A cells or after knocking out *DDR1* with two independent sgRNAs. **f** Phalloidin-stained secondary organoids from parental or *DDR1* knocked-out MCF10A cells. Scale bar = 100 µm. **g** Quantification of organoids from (**f**). P(Ctrl vs. sg1) = 2.28 × 10^−6^; P(Ctrl vs. sg2) = 7.56 × 10^−7^ (ordinary one-way ANOVA with Dunnett’s multiple comparisons test). Data were derived from *n* = 4 independently analyzed gels for each treatment group with *n* = 3 fields sampled per gel. **h** Quantification of secondary organoid development with or without DDR1 inhibition. *P* = 8.2 × 10^−5^ (two-sided Student’s *t*-test). Data were derived from *n* = 4 independently analyzed gels. For all bar graphs in this figure, data are presented as mean values ± SD. *** indicates *P* ≤ 0.001; **** indicates *p* ≤ 0.0001. Source data are provided as a Source data file.

When seeded as single cells in 3D matrix hydrogels, human breast epithelial cells from patient reduction mammoplasty specimens can regenerate an entire functional ductal-lobule unit^7^ (Fig. 1a). This occurs in a stepwise manner whereby single stem cells proliferate to form small spheroids before undergoing multilineage differentiation into organoids that initiate branching on day 10–13. Ducts then undergo budding and lobule formation by day 18 and continue to mature for an additional 10 days^7^ (Fig. 1a). When clusters of cells from partially-dissociated human breast tissue (organoids) are seeded in hydrogels, they regenerate complex ductal and lobular structures that closely resemble the functional TDLU present in the human breast^7^ (Fig. 1a). The kinetics of organoid regeneration is accelerated compared to single MECs, with initiation of branching typically occurring within the first 4 days and lobule maturation around days 10–12^7^ (Fig. 1a). Similarly, single cells from the human breast MCF10A cell line can give rise to complex ductal, lobular, and ductal-lobular tissues in a stepwise manner. Cells first proliferate to form small spheroids that, through branching and budding, form complex ductal/lobule structures by day 14–15^8^ (Fig. 1a). Importantly, when cultured in 3D collagen (in contrast to laminin-rich matrices such as Matrigel), MCF10A cells undergo multi-lineage differentiation toward the basal and luminal lineages^8^ (Supplementary Fig. 1a). Although generally developing similarly, MCF10A cells—unlike primary MECs—lose self-renewal activity during differentiation and maturation, and, consequently, are unable to regenerate secondary tissues. Inhibiting differentiation of MCF10A cells leads them to retain their self-renewal activity and the ability to reseed and regenerate secondary tissues^8^. Using this trait, we endeavored to identify potential regulators of tissue regeneration by screening for kinases that, when inhibited, would block differentiation. Kinases were selected due to their regulatory roles in cell differentiation, their interaction and cross-relationships with other signaling pathways implicated in cell fate decisions and importantly the existence of specific kinase inhibitors, enhancing the potential of findings being clinically relevant to breast cancer.

A custom pooled CRISPR sgRNA library containing 5070 guides targeting 507 human kinases was generated for this purpose^9^. The experimental design is depicted in Fig. 1b. We transduced MCF10A cells with the pooled sgRNA library and seeded infected cells or uninfected control cells in 3D hydrogels. Both sgRNA library-infected and control cells formed primary organoids, which were then dissociated, re-seeded, and allowed to form secondary organoids (Fig. 1b). The number of secondary organoids, indicative of self-renewal capability, increased dramatically in sgRNA library-infected cells, compared to uninfected control cells (Fig. 1c).

To identify kinases that inhibited self-renewal, we sequenced the transduced sgRNAs from the secondary organoids. An enrichment score for each kinase was calculated by comparing the sgRNAs reads in the secondary organoids to that of sgRNAs reads in the original transduced cells grown in 2D. 32 kinases were enriched in secondary organoids relative to pre-screened cells (Supplementary Table 1). We noted that several kinases (*FYN*^10^ www.ncbi.nlm.nih.gov/gene/2534, *MAPK7*^11^ www.ncbi.nlm.nih.gov/gene/5598, and *MAP3K7*^12^ www.ncbi.nlm.nih.gov/gene/6885) have been implicated previously in cellular differentiation, validating the robustness of the approach.

The most significantly enriched kinase gene identified in the screen was *DDR1* (www.ncbi.nlm.nih.gov/gene/780, Fig. 1d), encoding a collagen receptor. To validate the ability of *DDR1* loss to enable self-renewal, MCF10A cells were transduced with two additional independent sgRNAs against *DDR1* (Fig. 1e). Consistent with the screen results, cells lacking *DDR1* expression showed a significant increase in secondary organoid formation in 3D, indicative of retaining self-renewal (Fig. 1f, g). Furthermore, we used an independent small-molecule chemical approach to inhibit DDR1 kinase activity by blocking its autophosphorylation (DDR1i^13^). MCF10A cells treated with DDR1i exhibited the same phenotype of increased secondary organoid formation as those transduced with sgRNAs against *DDR1* (Fig. 1h).

To find out if the increase in secondary organoid formation was a result of increased proliferation induced by DDR1i treatment, we analyzed the effects of DDR1i on MCF10A cells cultured in 3D collagen gels. The results showed visible and measurable reduction in cell numbers following DDR1i (Supplementary Fig. 1b), confirmed by reduced dye dilution analysis of cell proliferation (Supplementary Fig. 1c). Cell cycle analysis indicated that DDR1i resulted in detectable increase in S and G2/M phase (Supplementary Fig. 1d). Given that this increase did not result in increase in cell numbers, we analyzed the effect of DDR1i on apoptosis using Annexin V assay and found no detectable effect (Supplementary Fig. 1e). Together, the results of these analyses indicate that increased proliferation is not a contributing factor to the increase in secondary structure formation. Thus, the increase in secondary organoid formation upon loss of DDR1 activity was confirmed both genetically and chemically, indicating that DDR1 is required for MCF10A stem cell differentiation.

**Fig. 2:**
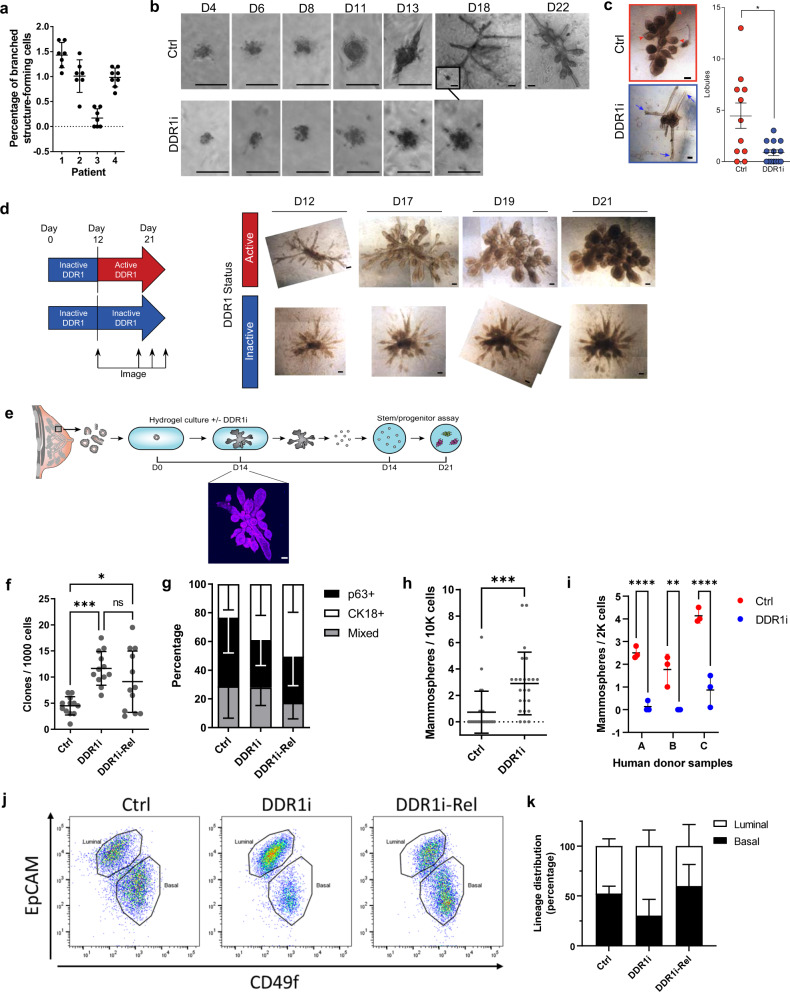
DDR1 is required for breast tissue regeneration. **a** Percentage of patient-derived single primary breast epithelial cells able to give rise to a branched stricture in 3D hydrogel. Data for patients 1–3 was derived from *n* = 7 gels each, and *n* = 8 gels for patient 4. Data are presented as mean values ± SD. **b** Development of single primary breast cells in hydrogels with or without DDR1i treatment. Inset in D18 top panel depicts image directly below (DDR1i, day 18) scaled to match top image (Ctrl, day 18). Results are representative of two independent repeats of this experiment. Scale bar = 100 µm. **c** Development of breast tissue organoids in hydrogels with or without DDR1i treatment (left) and quantification of lobules (right). Red arrowheads indicate lobules, blue arrows indicate ducts that terminated without a lobule. *P* = 0.0167 (two-sided Student’s *t*-test). Data were derived from *n* = 11 and *n* = 14 gels for the Ctrl and DDR1i-treated groups, respectively. Data are presented as mean values ± SEM. Scale bars = 100 µm. **d** Schematic of DDR1-inhibition and withdrawal experiments (left) and representative bright-field images after withdrawing DDR1i (right). Scale bars = 100 µm. **e** Schematic of stem/progenitor assay in primary breast organoids with or without DDR1i treatment. Representative image of vehicle-treated development after 14 days is shown below. Scale bar = 100 µm. **f** Clone formation by cells isolated from primary breast organoids with or without DDR1i treatment, and 2 days following release from DDR1 inhibition. Data were derived from *n* = 12 gels for each treatment group. P(Ctrl vs. DDR1i) = 0.0003; P(Ctrl vs. DDR1i-Rel) = 0.02 (ordinary one-way ANOVA with Tukey’s multiple comparisons test). Data are presented as mean values ± SD. **g** Percentage of clones composed exclusively of cells stained with either of the lineage markers CK18 and p63, or mixed. Data was derived from *n* = 12 gels for each treatment group. Data are presented as mean values ± SD. **h** Mammosphere formation by cells isolated from primary breast organoids cultured in hydrogel with or without DDR1i treatment. *P* = 0.0006 (two-sided Student’s *t*-test). Data were derived from *n* = 24 gels for each treatment group. Data are presented as mean values ± SD. **i** Mammosphere formation by primary breast cells cultured in suspension culture with or without DDR1i treatment. *P* = 8.8 × 10^−5^, 0.001 and 3 × 10^−6^ comparing Ctrl and DDR1i groups, for patients A, B, and C, respectively (ordinary two-way ANOVA with Sidak’s multiple comparisons test). Data were derived from *n* = 3 gels per patient for each treatment group. Data are presented as mean values ± SD. **j** Flow-cytometry dot-plot analysis of EpCAM and CD49f expression on cells from primary breast organoids grown in hydrogels for 14 days without or with DDR1i treatment, or released from DDR1 inhibition for the last 2 days. **k** Graph depicts cell population percentages under each condition. Data were derived from *n* = 2, *n* = 4, and *n* = 3 gels for the Ctrl, DDR1i and DDR1i-Rel groups, respectively. Data are presented as mean values ± SD. * Indicates *p* < 0.05; ** indicates *p* < 0.01; *** indicates *p* < 0.001; **** indicates *p* < 0.0001. Source data are provided as a Source data file.

To extend these findings to primary cells, primary MECs isolated from reduction mammoplasty patient tissue were seeded as single cells or as cell clusters (organoids) into 3D hydrogels and treated with DDR1i. The efficiency of branched structure formation from single cells seeded in hydrogels varies between donor samples and is around a frequency of ~1% of the total number of cells seeded (Fig. 2a). Based on this frequency, when seeding 4 gels with 1500 cells/gel, ~60 structures are expected to develop in total. However, when DDR1i was added to the media, no branched structures were identified in any of the gels seeded. Instead, single MECs formed small spheroids but failed to undergo multilineage differentiation and failed to initiate ductal elongation and lobule formation (Fig. 2b). When seeded as organoids in hydrogels in the presence of DDR1i, simple ducts formed but failed to initiate budding or form lobules, and TDLUs did not regenerate (Fig. 2c). To determine whether the block in TDLU regeneration could be reversed, organoids were cultured for 21 days: the first 12 days in the presence of DDR1i, and the last 9 days in the absence of DDR1i. Indeed, release from DDR1 inhibition led to full TDLUs formation (Fig. 2d) suggesting that DDR1 inhibition does not lead to an irreversible block in tissue regeneration. Together, these findings suggest that DDR1 activity is important for ductal elongation during early breast tissue regeneration, and for TDLU formation from the elongated ducts.

### DDR1 is required for bi-lineage differentiation

The block in tissue regeneration by inhibiting DDR1 could be due to defects in stem/progenitor cells and/or defects in differentiation. To assess this, primary tissues were cultured in 3D hydrogels in the presence or absence of DDR1i for 14 days or released from DDR1 inhibition for the last 2 days in culture (days 12–14, DDR1i-Rel). Cells were dissociated from the hydrogels and MECs were examined for stem/progenitor cell activity by colony-forming unit (CFU) assay and mammosphere assay (Fig. 2e–h). The CFU assay calculates the number of stem and progenitor cells by quantifying the number of unipotent (luminal or basal only) or bi-potent (mixed luminal and basal) colonies that form after seeding at low density. DDR1 inhibition led to >2-fold increase in the proportion of colony-forming cells (*P* ≤ 0.001, Fig. 2f). Immuno-staining of the clones that formed for the lineage markers CK18 and p63 revealed no significant changes in clone lineages between the control and DDR1i-treated groups. An increased percentage of luminal clones (CK18^+^) was noted two days following release from DDR1 inhibition, compared with control (*P* = 0.002, Fig. 2g). The mammosphere assay estimates the number of stem cells based on their ability to form colonies under non-adherent conditions (in suspension). Cells isolated from hydrogel structures that were under DDR1i treatment formed significantly more mammospheres compared with cells from untreated control hydrogels (3.6-fold, *P* = 0.0005, Fig. 2h). DDR1i treatment of primary human breast cells seeded directly in suspension culture (never cultured in hydrogels) resulted in dramatically diminished mammosphere formation compared with untreated controls, supporting inhibition of stem cell proliferation/differentiation (Fig. 2i). Together, these results indicate that the failure to form TDLUs was not due to a depletion in stem or progenitor numbers, but rather due to inhibition of their function.

The percentages of basal cells (CD49f^+^/EpCAM^−/low^) and luminal cells (CD49f^−/low^/EpCAM^+^) from 3D tissues grown in hydrogels in the presence or absence of DDR1i, or released from DDR1i for the last 2 days in culture (DDR1i-Rel), were analyzed by flow cytometry (Fig. 2j). Despite a trend toward increased percentage of luminal cells, DDR1i treatment did not result in statistically significant differences in the luminal/basal lineage ratio, similarly to the findings from the CFU assay. These results indicate that DDR1 may have a minor effect on the luminal/basal ratio of cells but does not significantly change the lineage balance.

**Fig. 3:**
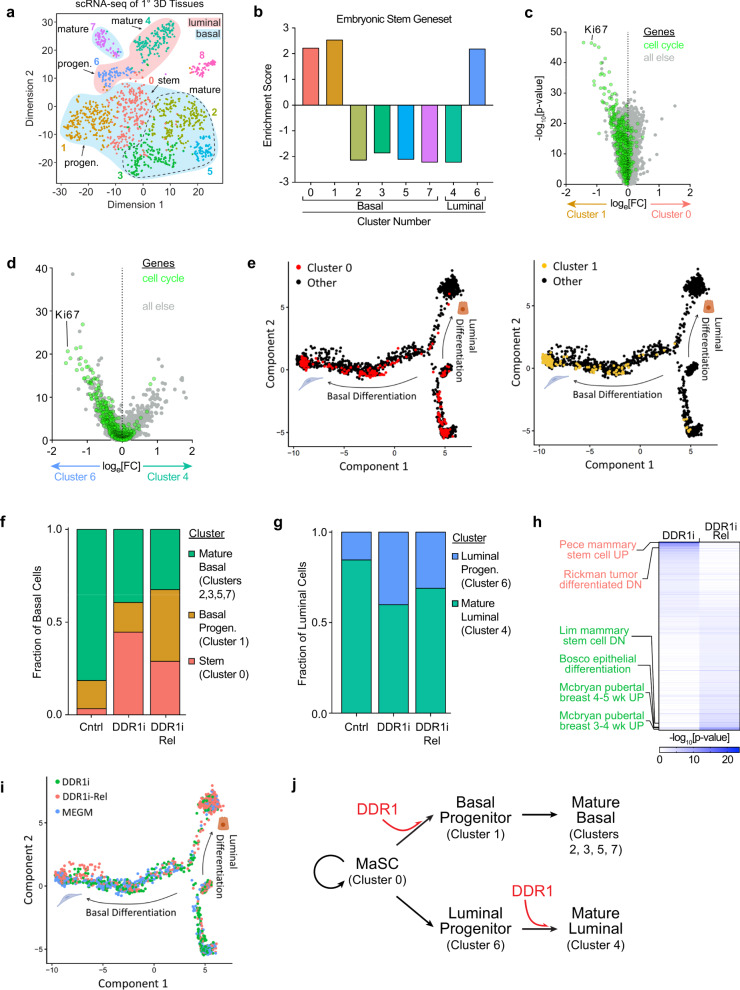
scRNA-seq of primary tissues shows cell-state changes mediated by DDR1. **a** scRNA-seq data from all cells (*n* = 1467) from patient-derived hydrogel-grown tissues projected onto two dimensions using t-SNE on the top eight principal components across 7193 variable genes. **b** Bar chart of an embryonic stem cell gene set enrichment in each of the epithelial clusters. **c**, **d** Volcano plot visualizing differential gene expression between clusters 0 and 1 (**c**) and between clusters 4 and 6 (**d**). Cell cycle genes are highlighted in green. Ki67 (*MKI67*, www.ncbi.nlm.nih.gov/gene/4288) is labeled individually. The likelihood ratio test was used, p-values not corrected for multiple tests. **e** Inferred lineage relationships of all cells (black) were projected onto two dimensions as basal and luminal differentiation trajectories, using Monocle. Cluster 0 (red; left panel) and cluster 1 (yellow; right panel) cells are highlighted. **f**–**g** Stacked bar charts indicating the distribution of basal (**f**) and luminal (**g**) clusters in control, DDR1i, and DDR1i Rel tissues. **h** Heatmap of enriched gene sets using the Broad Institute’s MAigDB dataset with the hypergeometric test (FDRs were calculated) on DDR1i and DDR1i Rel tissues. **i** Cells treated with DDR1i, released from DDR1i and untreated controls projected onto basal and luminal differentiation trajectories, using Monocle. **j** Proposed model for the role of DDR1 in mammary epithelial cell differentiation. Source data are provided as a Source data file.

To better understand the role of DDR1 in differentiation, we performed scRNA-seq using Seq-Well^14^ to generate a comparative cellular atlas at single-cell resolution on human breast tissues grown for 14 days in 3D hydrogels under three different conditions: 1) vehicle-treated control, 2) DDR1-inhibited (DDR1i), and 3) DDR1-inhibited for 12 days followed by release from inhibition for 2 days (DDR1i Rel). We first examined the molecular heterogeneity in 14-day human breast tissue outgrowths. Unsupervised analysis of 1467 cells, with greater than 500 genes detected per cell, revealed nine distinct clusters corresponding to different cell states in 3D human breast tissues (Fig. 3a).

Using unbiased clustering analysis, spatial reconstruction of single cell data, and integrated analysis across all three conditions, we classified cells into 8 epithelial clusters based on a unique group of expressed genes (Fig. 3a). Table 1 summarizes the evidence for cell-state classification of each epithelial cluster. Analysis using genes encoding epithelial lineage-specific cytokeratins (Luminal: *KRT8* www.ncbi.nlm.nih.gov/gene/3856, *KRT18* www.ncbi.nlm.nih.gov/gene/3875, *KRT19* www.ncbi.nlm.nih.gov/gene/3880; Basal: *KRT14* www.ncbi.nlm.nih.gov/gene/3861, *KRT5* www.ncbi.nlm.nih.gov/gene/3852) revealed six of the clusters were of the basal lineage (clusters 0, 1, 2, 3, 5, 7) while the remaining two clusters were of the luminal linage (clusters 4 and 6; Supplementary Fig. 2a, b). Gene set enrichment analysis (GSEA) for an embryonic stem cell geneset^15^ further defined clusters 0 and 1 as undifferentiated stem/progenitor cells within the basal lineage and cluster 6 as undifferentiated stem/progenitor cells within the luminal lineage (Fig. 3b). Analysis of differential expression of a cell cycle geneset^16^ between clusters 0 and 1 and clusters 4 and 6 revealed that clusters 1 and 6 displayed high scores for proliferation signature, indicating that these clusters were active progenitor cells (Fig. 3c, d). The remaining clusters (basal clusters 2, 3, 5, 7, and luminal cluster 4) consisted of more mature lineage-restricted differentiated cells. Luminal cluster 4 expressed high levels of *LTF* (encoding the protein Lactotransferrin, www.ncbi.nlm.nih.gov/gene/4057), consistent with mature lobular luminal cells (Supplementary Fig. 2c).

**Table 1:** Mammary cell states corresponding to scRNA-seq clusters.

Cluster	Lineage	Stem cell genes	Proliferation	Differentiation potential	Cell state
0	Basal (*KRT14*^+^)	Upregulated	−	Lum + Basal	Bipotent stem
1	Basal (*KRT14*^+^)	Upregulated	+	Basal	Basal progenitor
2,3,5,7	Basal (*KRT14*^+^)	Downregulated	−	Basal	Mature basal
4	Luminal (*KRT8*^+^)	Downregulated	−	Luminal	Mature luminal
6	Luminal (*KRT8*^+^)	Upregulated	+	Luminal	Luminal progenitor

Summary of cluster identification using: lineage information derived from cytokeratin expression; stem cell gene expression enrichment determined by GSEA; proliferative potential based on Ki67 expression and cell cycle gene enrichment; and differentiation potential determined by the presence of cells within a cluster along lineage reconstruction paths by Monocle. Alongside the summaries of cluster characteristics are the identities of the mammary cell states to which these clusters were assigned.

To understand the lineage relationships between cell clusters, we constructed a pseudotemporally ordered tree using Monocle. This analysis revealed that many cluster 0 cells were undifferentiated or along the basal differentiation trajectory with a minority along the luminal differentiation trajectory. Cluster 1 cells were farther along the basal differentiation trajectory and only a minority were undifferentiated. (Fig. 3e). Likewise, cluster 6 was restricted to the luminal lineage, giving rise to cluster 4, which scored further along the luminal differentiation dimension (Supplementary Fig. 2d).

Together, these results show that human breast tissues that grow in 3D hydrogels retain a cellular hierarchy including stem cells, lineage-restricted luminal and basal progenitors, and mature luminal and basal cells.

Having assessed the epithelial hierarchy and their linear relationship to differentiated cells in normal human breast tissue, we next asked how cell states were affected by DDR1 activity. Inhibition of DDR1 activity blocked the differentiation of stem cells, as evidenced by a dramatic increase in the proportion of bipotent stem cells, with a concomitant decrease in the number of mature basal cells (Fig. 3f). In addition, inhibition of DDR1 activity resulted in a significant increase in the fraction of proliferative luminal progenitors, indicating that in the absence of DDR1, luminal cells were trapped in a proliferative progenitor state but failed to differentiate into mature luminal cells (Fig. 3g). Upon release from DDR1 inhibition for the final two days of culture, expression levels of genes associated with mammary epithelial differentiation and early development were enriched (Fig. 3h). In addition, we observed a trend toward an increase in mature luminal cells with concomitant decrease in luminal progenitors, as well as a decrease in stem cell number concomitant with increase in the fraction of basal progenitors (Fig. 3f, g).

We analyzed the effect of DDR1i and release on the position of cells along the pseudotemporal trajectories of luminal and basal differentiation. DDR1 inhibition resulted in a block of cell progression along both trajectories, and accumulation of undifferentiated cells. Release of DDR1 inhibition released this block and allowed progression of cells toward both the luminal and basal differentiation axes (Fig. 3i).

Collectively, these results indicate that DDR1 is required for the differentiation of basal progenitors from bi-potent stem cells, as well as the differentiation of mature luminal cells from luminal progenitor cells (Fig. 3j).

### DDR1 promotes luminal differentiation by activating Notch1

**Fig. 4:**
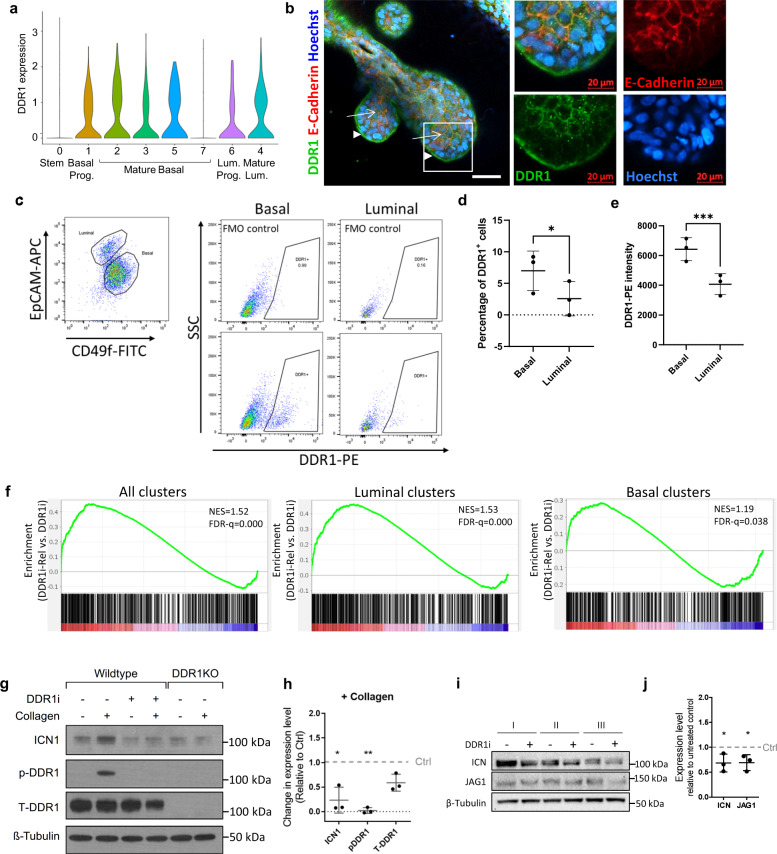
DDR1 signaling activates Notch1 to drive luminal differentiation. **a** Violin plot showing the distribution of *DDR1* expression in epithelial clusters. **b** Immunofluorescent staining of DDR1 (green), E-cadherin (red) and Hoechst nuclear staining (blue) in patient-derived hydrogel-grown tissues. Arrows: basal expression of DDR1. Arrowheads: luminal expression of DDR1. Right panels depict enlargement of region in white rectangle. Results are representative of three independent repeats of this experiment. **c** Flow cytometry analysis of primary breast tissue cultured in 3D hydrogels for 12 days. Basal and luminal cells were identified based on EpCAM and CD49f expression and further analyzed for DDR1 expression. **d** The percentage of DDR1^+^ cells was compared between the basal and luminal populations. *P* = 0.03 (two-sided Student’s *t*-test). **e** DDR1 signal intensity was compared between the basal and luminal populations. *P* = 0.0008 (two-tailed Student’s *t*-test). Data were derived from *n* = 3 independent patient samples. Data are presented as mean values ± SD. **f** GSEA plots depicting enrichment of a Notch1 target gene set among genes that are overexpressed in cells from DDR1i Rel tissues compared to DDR1i tissues. Plots depict enrichment across all clusters, luminal clusters only and basal clusters only. Normalized enrichment score (NES) and false discovery rate q-value (FDR-q) are indicated. **g** Western blot for phospho-DDR1, total-DDR1, and cleaved Notch1 (ICN1) under the indicated conditions. β-tubulin was used as a loading control. **h** Graph depicts quantification of *n* = 3 biological repeats of western blot seen in (**g**), comparing DDR1i treated samples with untreated controls in the presence of collagen. P(ICN1 vs. Ctrl) = 0.0373, P(pDDR1 vs. Ctrl) = 0.0012 (two-sided Student’s *t*-tests). Data are presented as mean values ± SD. **i** Western blot for cleaved Notch1 (ICN1) and Jagged-1 in separately obtained MCF10A cell lines cultured in 3D collagen in the presence or absence of DDR1i. β-tubulin was used as a loading control. **j** Graph depicts quantification of *n* = 3 biological repeats seen in (i), comparing DDR1i treated samples with untreated controls. P(Ctrl vs. ICN1) = 0.039, P(Ctrl vs. JAG1) = 0.028. (two-sided Student’s *t*-test)* indicates *P* < 0.05. ** indicates *P* < 0.01. *** indicates *P* < 0.001. Data are presented as mean values ± SD. * indicates *P* < 0.05. Source data are provided as a Source data file.

Recognizing that DDR1 activity promoted differentiation in more than one mammary lineage, we aimed to identify how DDR1 exerts its effect on different cell types. First, we looked to characterize DDR1 expression in the different cell types. Using scRNA-seq gene expression analysis and confocal imaging of immunofluorescence-stained 3D structures in hydrogel, we found that DDR1 is expressed in both luminal and basal cells (Fig. 4a, b). In luminal cells, DDR1 is localized to cell surfaces in contact with adjacent luminal cells, but not to basolateral surfaces in contact with basal cells (Fig. 4b, arrows). In contrast, in basal cells DDR1 is localized to the basolateral surfaces in contact with the ECM, but not on the apical surfaces in contact with luminal cells (Fig. 4b, arrowheads). Flow cytometry analysis of DDR1 expression in luminal and basal cells, as identified based on CD49f and EpCAM expression, showed that an average of 7% of basal cells are DDR1^+^ compared with only 2.5% of luminal cells (*P* < 0.05, Fig. 4c, d). Additionally, the signal intensity of DDR1 was significantly higher in basal compared with luminal cells (*P* < 0.001, Fig. 4e). DDR1 on basal cells can be activated by matrix collagen, but DDR1 sequestered to E-cadherin expressing cell–cell interfaces on luminal cells cannot^17^, raising the possibility that basally-expressed DDR1 acts indirectly on luminal cells to drive luminal cell maturation and lobular growth.

As Notch signaling is required for luminal cell and lobular differentiation^18–20^ and DDR1 signaling has been shown in other contexts to activate Notch signaling^21^, we asked whether DDR1 activation results in upregulation of Notch signaling. GSEA analysis revealed that Notch1 target genes^22^ are significantly enriched among genes that are upregulated following release from DDR1 inhibition (DDR1i Rel tissues; Fig. 4f). Moreover, the enrichment of Notch1 target genes was higher in luminal cells compared with basal cells, making the luminal lineage the main contributor to the overall enrichment of Notch1 target genes (Fig. 4f). Expression of the Notch1 target genes *MAP3K8* (www.ncbi.nlm.nih.gov/gene/1326), *KRT23* (www.ncbi.nlm.nih.gov/gene/25984) and *KLF6* (www.ncbi.nlm.nih.gov/gene/1316) was mapped to the clusters identified by the scRNA-seq, and was highly expressed in the mature luminal cluster 4, further supporting for the activation of Notch1 target genes in the luminal lineage (Supplementary Figs. 3a–c). Together, these findings suggest that DDR1 stimulation at the matrix-cell interface triggers Notch1 signaling in adjacent luminal cells.

To directly test whether DDR1 activation by collagen leads to Notch1 activation, we stimulated MCF10A cells in 2D culture with collagen and assessed DDR1 and Notch1 activity. DDR1 activation and phosphorylation was induced upon collagen stimulation as was activation of Notch1 cleavage (Fig. 4g). Densitometry quantification showed that when stimulated with collagen, DDR1 inhibition leads to significant reduction in Notch1 activation, as evidenced by decreased levels of cleaved Notch1 (Fig. 4h). Similarly, collagen failed to induce Notch1 cleavage in cells lacking *DDR1* (DDR1KO, Fig. 4g). In addition, we analyzed the effect of DDR1 inhibition on Notch1 signaling in a 3D context (collagen gels) revealing reduced expression of both cleaved Notch1 (ICN1) and the Notch1 ligand Jagged-1 following DDR1 inhibition (Fig. 4i, j). Together, these results demonstrate that DDR1 activation by collagen activates Notch1 signaling.

Next, we examined whether the failure in TDLU formation upon DDR1 inhibition was due to the lack of Notch activation. We treated organoids seeded in 3D hydrogels with a γ-secretase inhibitor (GSI), which prevents Notch cleavage and nuclear translocation^23^. Indeed, the presence of GSI led to a failure of TDLU formation, phenocopying the defect due to inhibition of DDR1 (Supplementary Fig. 3d). Taken together, these findings indicate that breast tissue regeneration in 3D culture involves collagen stimulation of DDR1 in basal cells, leading to activation of Notch1 signaling in adjacent luminal cells, driving luminal differentiation and TDLU formation.

### Jagged-1 mediates ECM-basal cell signals driving luminal differentiation

**Fig. 5:**
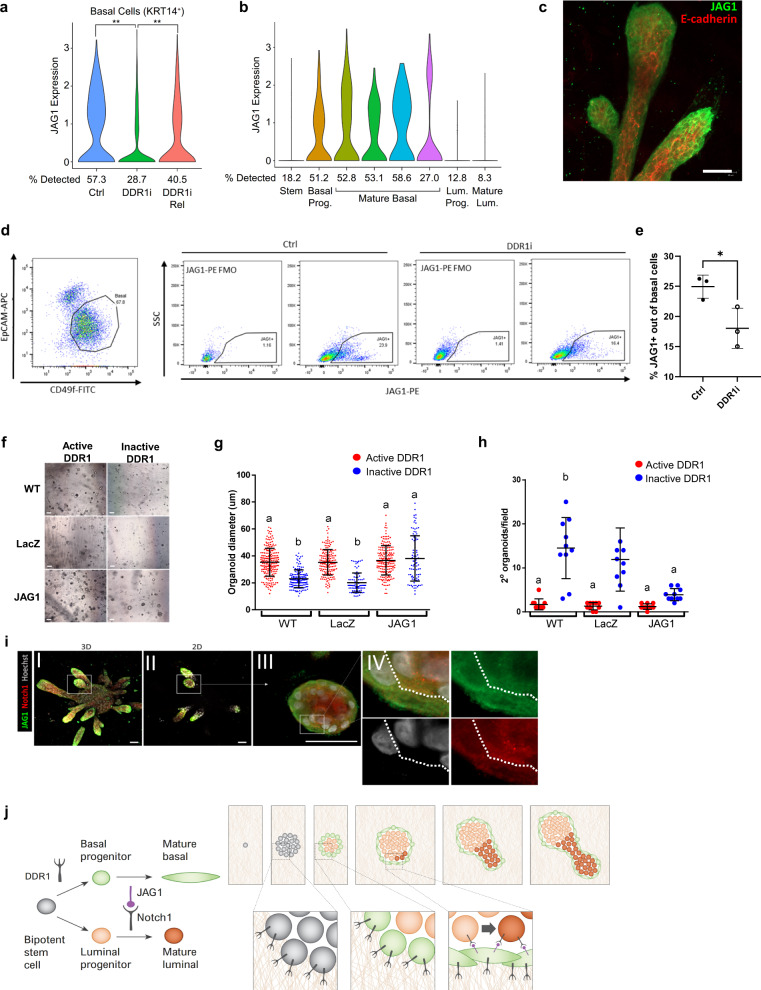
Jagged-1 mediates signal from ECM-sensing basal cells to drive luminal cell differentiation. **a** *JAG1* expression in basal cells from control, DDR1i, and DDR1i Rel tissues. P(DDR1iR vs Ctrl) < 2.5e−14, p(DDR1i vs Ctrl) < 7e−20, p(DDR1iR vs DDR1i) = 0.38. The likelihood ratio test (FDR) was used, not corrected for multiple testing. **b** *JAG1* expression among epithelial clusters. **c** Immunofluorescent staining of Jagged-1 (green) and E-cadherin (red) in 3D-cultured patient-derived tissues. Results are representative of three independent repeats of this experiment. **d**, **e** Flow cytometry analysis of primary tissue in 3D hydrogels treated with DDR1i for 12 days. Basal cells were identified based on EpCAM and CD49f expression and further analyzed for Jagged-1 expression (**d**). The percentage of Jagged-1^+^ cells (**e**) was compared between control and treated groups. Data were derived from *n* = 3 independent patient samples. *P*-value = 0.035, two-sided Student’s *t*-test. * Indicates *P* ≤ 0.05. Data are presented as mean values ± SD. **f**–**g** Development of MCF10A cells (WT, overexpressing *LacZ* or overexpressing *JAG1*) in 3D collagen with or without DDR1i. Representative images shown in (**f**), diameter of organoids shown in (**g**). Organoid diameter was measured for each treatment group. *n* = 206 and *n* = 145 organoids were measured in the WT group cultured under active or inactive DDR1, respectively. *n* = 166 and *n* = 92 organoids were measured in the LacZ group cultured under active or inactive DDR1, respectively. *n* = 183 and *n* = 92 organoids were measured in the JAG1 group cultured under active or inactive DDR1, respectively. Letters over graph bars indicate statistically different groups, *P* = 3 × 10^−13^ (One-way ANOVA with Tukey’s multiple comparisons test). Data are presented as mean values ± SD. **h** Quantification of secondary organoid development by cells isolated from structures formed by WT, *LacZ*-overexpressing, or *JAG1*-overexpressing MCF10A cells with or without DDR1i treatment. Data were derived from *n* = 10 fields analyzed for each treatment group. Letters over graph bars indicate statistically different groups, *P* ≤ 0.001 (two-way ANOVA with Tukey’s multiple comparisons test). Data are presented as mean values ± SD. **i** Immunofluorescent staining of Jagged-1 (green), Notch1 (red) and Hoechst (gray) in 3D-cultured patient-derived tissues. (I) 3D reconstruction of a Z-stack captured by confocal microscopy. (II) a single 2D plane across lobule framed by rectangle in (I). (III) high-power (×60) image of lobule framed by rectangle in (I) and (II). (IV) Enlargement of area framed by rectangle in (III). **j** Model for the role of DDR1, Jagged-1, and Notch1 within the mammary differentiation hierarchy (left) and visual depiction of how tissue regeneration and morphogenesis is controlled by these factors through spatially regulated cell fate decisions (right). Scale bars = 50 µm. * Indicates *p* < 0.05; ** indicates *p* < 0.01. Source data are provided as a Source data file.

We next inquired how DDR1 stimulation on basal cells leads to Notch1 activation on luminal cells. Notch signaling is a conserved pathway that regulates cell fate decisions and is initiated by binding of a transmembrane ligand (Jagged (JAG) or Delta-like (DLL)) expressed on one cell to a Notch receptor expressed on an adjacent cell. We examined the expression of the Notch1 ligands *JAG1* (www.ncbi.nlm.nih.gov/gene/182), *JAG2* (www.ncbi.nlm.nih.gov/gene/3714)*, DLL1* (www.ncbi.nlm.nih.gov/gene/28514)*, DLL3* (www.ncbi.nlm.nih.gov/gene/10683) and *DLL4* (www.ncbi.nlm.nih.gov/gene/54567) in basal cells following DDR1 inhibition and release and found that *JAG1* expression is downregulated following DDR1 inhibition and upregulated following release (Fig. 5a). The other Notch1 ligands were minimally expressed (*JAG2, DLL1, DLL3*, and *DLL4*), and their expression did not change upon DDR1 inhibition and release (Supplementary Fig. 4). scRNA-seq revealed that *JAG1* is expressed exclusively in basal clusters, including basal progenitors and mature basal cells, but not in MaSC (Fig. 5b). As expected, based on previous findings in-vivo^24^, immunofluorescent staining confirmed the basal location of Jagged-1 in primary breast tissue cultured in 3D hydrogels (Fig. 5c). To confirm the effect of DDR1 inhibition on Jagged-1 expression in basal cells, we treated primary breast tissue in 3D cultures with DDR1 inhibitor for 12 days and used flow cytometry to analyze Jagged-1 expression levels in the basal population, as defined by EpCAM and CD49f expression. The results confirmed the scRNA-seq finding that DDR1 inhibition leads to downregulation of Jagged-1 in the basal population (Fig. 5d, e).

The finding that DDR1 regulates Jagged-1 expression in basal cells, while also regulating Notch1 signaling in adjacent luminal cells, suggested the possibility that Jagged-1 acts to transduce DDR1 signaling from the basal to the luminal layer, driving the differentiation of luminal progenitors to mature luminal cells via the Notch1 signaling pathway.

To establish whether Jagged-1 mediates the DDR1-induced differentiation signal, we tested whether *JAG1* overexpression could rescue the block in differentiation induced by DDR1 inhibition. We created an MCF10A cell line that stably overexpresses *JAG1* (MCF10A/*JAG1*) as well as a control cell line (MCF10A/*LacZ*, www.ncbi.nlm.nih.gov/gene/945006) (Supplementary Fig. 5a). 3D culture in collagen of the MCF10A/*JAG1* in the presence or absence of DDR1i revealed that DDR1 inhibition resulted in downregulation of Jagged-1, which nevertheless remained 5-fold higher compared with DDR1i-treated MCF10A/*LacZ* control cells (Supplementary Figs. 5b, c). Wild-type (WT) MCF10A, MCF10A/*LacZ,* and MCF10A/*JAG1* were cultured in collagen gels in the presence or absence of DDR1i and allowed to form 3D organoids. We noted that DDR1 inhibition resulted in significantly smaller organoids in WT MCF10A and MCF10A/*LacZ*, but not in MCF10A/*JAG1*, indicating that Jagged-1 overexpression enables normal organoid growth in the absence of active DDR1 (Fig. 5f, g). Despite their smaller size, DDR1i-treated organoids formed by WT MCF10A and MCF10A/*LacZ*, but not MCF10A/*JAG1*, had a higher percentage of DNA-replicating cells, as evidenced by EdU incorporation, compared with untreated organoids (Supplementary Fig. 5d, f). This demonstrates that active DDR1 enables cell differentiation, resulting in larger organoids comprised mainly of terminally differentiated, non-cycling cells. DDR1 inhibition arrested cell differentiation, resulting in smaller organoids with a higher percentage of DNA-replicating progenitor cells, an effect that was eliminated by *JAG1* overexpression.

Finally, to assess their self-renewal potential, the organoids were dissociated into single cells and re-seeded in a second set of collagen gels. The second set of gels was not treated with DDR1i. As expected, DDR1i-treated WT MCF10A and MCF10A/*LacZ* cells formed significantly more secondary organoids compared with untreated controls, while MCF10A/*JAG1* cells formed very few organoids regardless of DDR1i treatment (Fig. 5h), confirming that *JAG1* overexpression rescued the DDR1i-induced differentiation arrest.

We were able to localize the region of potential Jagged-1-Notch1 interaction in 3D tissues by immunofluorescence staining. 3D reconstruction of regenerating TDLUs revealed the interface of Jagged-1-expressing basal cells and Notch1 expressing luminal cells, constituting the region in which the DDR1-Jagged-1-Notch1 signaling axis can take place to drive differentiation (Fig. 5i).

Taken together, these data show that DDR1 is required for TDLU regeneration through the differentiation of bipotent mammary stem cells into lineage-committed basal progenitors (Fig. 5j). Basal cells express the Notch1 ligand Jagged-1, which is required for activation of Notch1 signaling in luminal progenitor cells. This activation of luminal Notch1 signaling, in turn, induces the maturation of luminal progenitors into mature luminal cells, driving TDLU growth (Fig. 5j).

## Discussion

Since stem cells were first identified, researchers have been working toward understanding how they can be used to repair or replace damaged tissue. Tissue regeneration in a 3D context is facilitated by spatially regulated cell fate decisions. In the breast, this process is further complicated by the presence of two epithelial lineages and the dynamic nature of tissue maturation and regression that accompany reproductive cycles. Using a biomimetic hydrogel scaffold, we describe here a mechanism by which stem cells regenerate the breast tissue in 3D by a spatially regulated re-population of the entire mammary cell hierarchy.

Other studies have described a profiling of human breast epithelial cells using clustering based on scRNA-seq^25,26^. Nguyen et al.^26^ profiled breast epithelial cells isolated from donated tissue. Despite differences in the methodology used for identification of cells clusters, both the analysis presented here of breast tissue cultured in 3D hydrogels as well as the analysis performed on uncultured breast tissue, identified similar populations: a stem-cell enriched population, mature basal cells, luminal progenitors and mature luminal cells. Several sub-clusters of basal cells were identified in both analyses, and MaSC proceeded to give rise to more mature basal populations and to luminal lineages on separate branches. Interestingly, another analysis recently identified a cluster expressing markers of both the luminal and basal lineages^25^, consistent with a “luminobasal” cell state, previously described in both normal breast^27^ and in breast tumors^28^. In our analysis, the genes enriched in this cluster are most consistent with cluster 4 (mature luminal), and it will be interesting to further investigate what regulates a luminobasal cell state, and what are its potential cell fates. Broadly, the parallels between cell clustering of breast tissue and that of 3D breast organoids indicate that the latter maintain the main cell states and lineages that exist in vivo.

Investigating the mechanistic control of stem cell fate in the developing breast is complicated due to lack of a suitable model. Extracellular and microenvironmental signals are key in epithelial morphogenesis, but the stroma of the human and the mouse are very different, making the mouse a less-than ideal model in which to study breast morphogenesis. A 3D model of primary human breast development in a biomimetic matrix offers an opportunity to study human breast development. While the 3D organoid model is limited in its ability to recapitulate all aspects of the human breast environment, it allows us to identify mechanisms directly relevant to the human physiology, which can then be further explored.

DDR1 activation by ECM collagen facilitates the differentiation of stem cells to basal cells, giving rise to the basal progenitors and mature basal cells that encapsulate the growing structure. DDR1 on inner cells that are not in contact with the ECM is not activated, and they assume luminal identity. The DDR1-expressing encapsulating cells upregulate the Notch ligand Jagged-1, which activates Notch1 on adjacent inner cells, promoting their differentiation to mature luminal cells, resulting in budding, branching, and development of TDLU: the functional unit of the breast glandular epithelium.

The mechanism downstream of DDR1 by which it regulates Jagged-1 expression is unclear, but several lines of evidence point to a reciprocal regulation between the two. Studies in liver show extensive colocalization and co-IP of Jagged-1 and DDR1^29^. In the breast epithelium, we also see co-expression of Jagged-1 and DDR1 on basal cells. In addition, DDR1 expression is increased in livers of mice heterozygous for *JAG1* conditional/null alleles compared with littermate controls^29^, indicating that the regulation of expression can occur in both directions. Interestingly, in our study we created a cell line that stably expresses *JAG1* under the CMV promoter, which when treated with DDR1i exhibited decreased Jagged-1 expression (Supplementary Fig. 5b), suggesting that the mechanism of DDR1 regulation of Jagged-1 expression occurs post-transcription. Coincidentally or not, both Jagged-1 and DDR1 were shown to be direct targets of a single miRNA, *miR‐199b‐5p* (www.ncbi.nlm.nih.gov/gene/406978), demonstrating a common post-transcriptional mechanism for these two proteins^30^, but how they co-regulate each-other’s expression remains elusive.

Given the prevalence of collagen in extracellular matrices across epithelial tissues, the regeneration mechanism described here may facilitate advances in tissue engineering strategies to repair and replace other complex glandular tissues.

## Methods

### Ethics statement

Primary tissues that would otherwise have been discarded as medical waste following surgery were obtained in compliance with all relevant laws, using protocols approved by the institutional review board at Maine Medical Center and Tufts Medical Center. All tissues were anonymized before transfer and could not be traced to specific patients; for this reason, this research was provided exemption status by the Committee on the Use of Humans as Experimental Subjects at the Massachusetts Institute of Technology, and at Tufts University Health Sciences (IRB# 13521). All patients enrolled in this study signed an informed consent form to agree to participate in this study and for publication of the results.

### Cell culture and preparation of patient-derived tissue

MCF10A cells were obtained from ATCC (CRL-10317) and cultured in MEGM (Lonza CC-3150) supplemented with 100 ng/ml cholera toxin (Sigma-Aldrich), 1× GlutaMax, and 1× Penicillin and Streptomycin (Gibco). Reduction mammoplasty tissue samples were mechanically dissociated and then incubated with 3 mg/ml collagenase (Roche Life Science, Indianapolis, IN, USA) and 0.7 mg/ml hyaluronidase (Sigma-Aldrich, St. Louis, MO, USA) at 37 °C overnight. Epithelial clusters were disrupted by trituration, washed, and depleted for fibroblasts. For culture of single primary cells in hydrogels, epithelial clusters were further digested in 0.25% trypsin-EDTA for 4 min at 37 °C followed by 5 mg/mL Dispase II (Roche) with 0.1 mg/mL DNase I (Sigma) for 2 min, and then filtered through a 40 mm mesh filter to yield a single-cell suspension.

### 3D culture

MCF10A cells were resuspended in 1.25 mg/ml rat tail collagen I (Corning) in PBS, brought to pH 7.3 with 0.1 N NaOH solution. Primary cells were resuspended in 1.7 mg/ml collagen I (Corning), 10 μg/ml hyaluronic acid (Sigma), 40 μg/ml laminin (Thermo Fisher Scientific), and 20 μg/ml fibronectin (Thermo Fisher Scientific), pH 7.3. Collagen gels and hydrogels were produced in a four-chamber slide (Corning) as a mold and incubated 1 h at 37 °C for polymerization before adding medium^7,8^.

### CRISPR–Cas9 screen

Cells were transduced with Cas9 (50661, Addgene) and, subsequently, with a kinase targeting sgRNA library (51044, Addgene) as previously described^9^. pCW-Cas9 was a gift from Eric Lander & David Sabatini (50661, Addgene). Human CRISPR enriched pooled library was a gift from David Sabatini & Eric Lander (51044, Addgene). Cells were then seeded and grown in collagen matrices for 10 days. Then the collagen pads were collected and incubated in 100 μg/ml collagenase in PBS at 37 °C for 10 min. The structures were collected by centrifugation (300 × *g*, 5 min), trypsinized with 0.25% trypsin for 20–25 min at 37 °C. Cells were counted in trypan blue, spun down (300 × *g*, 5 min), and resuspended in MCF10A media; 7500 living cells were reseeded into a new collagen pad. After growth in the new collagen pads for 7 days, collagen pads were harvested to collect genomic DNA, sgRNA inserts were PCR amplified in a nested PCR and the resulting libraries were submitted for sgRNA barcode sequencing with a HiSeq 2500. Sequencing reads were aligned to the sgRNA library, and the abundance of each sgRNA was calculated for each sample by normalizing sgRNA reads to total reads from the sample. RIGER-E was used to calculate an FDR corrected significance via the “second best” metric^31^. sgRNA representation in secondary organoids was calculated by subtracting the log2 transformed sgRNA abundance in 3D by the log2 transformed sgRNA abundance in 2D.

### Proliferation and apoptosis assays

Cells were seeded in collagen gels and allowed to grow for 10 days with or without DDR1i (2 µM). For EdU proliferation assay, EdU was added on day 10 and gels were allowed to grow for two more days and fixed for analysis on day 12. EdU fluorescence detection was performed according to the manufacturer’s protocol (C10640, Thermo Fisher). The percentage of EdU^+^ cells per organoid was analyzed in 10 organoids of each group. For cell cycle analysis, cells were harvested on day 10 and labeled with DRAQ5 membrane permeable dye (65-0880-96, Invitrogen) at a concentration of 5 µM and analyzed using a flow cytometer (BD LSRII at the Tufts Flow Cytometry Core). Analysis of cell cycle phases was performed on FlowJo software v10.7 using the Dean-Jett-Fox model. For dye dilution analysis, CellTrace CFSE cell proliferation kit for flow cytometry was used (C34570, Thermo Fisher) according to manufacturer protocol. Cells were labeled with 5 µM CFSE immediately before embedding in collagen gels, and isolated and analyzed by flow-cytometry (as above) after 3 days in culture. For Annexin V assay, Dead Cell Apoptosis Kit with Annexin V FITC was used (V13242, Thermo Fisher) according to manufacturer protocol. For cell cycle, dye dilution and annexin V analyses, 3 samples were individually cultured, treated, isolated and analyzed in each treatment group.

### Colony and mammosphere assays

Patient-derived mammary tissues (*n* = 3) were grown in hydrogels for 14 days in the presence or absence of DDR1 inhibitor (2 μM; 5077, Tocris)^13^. An additional set of gels (*n* = 3) was cultured in the presence of DDR1i for 12 days, and in the absence of DDR1i for the last 2 days in culture. Tissues were then dissociated in 100 µg/ml collagenase in PBS at 37 °C for 10 min, collected by centrifugation (300 × *g*, 5 min), resuspended and incubated in 0.25% trypsin for 10 min at 37 °C, then washed and counted in trypan blue. Cells were seeded and cultured for one week (adherent colonies) or 2 weeks (mammospheres). Colony assay seeding density was 2500 cells/cm^2^. Mammosphere seeding density was 12,500 cells/well in 24-well ultra-low attachment plate. *n* = 12 wells were analyzed for colony assay, *n* = 24 wells for mammosphere assay. For analysis of DDR1i effect on mammosphere formation when applied directly to cells in suspension culture, patient-derived mammary tissues (*n* = 3) were dissociated to single cells and cultured in ultra-low attachment plates as described above.

### Lentivirus production and infection

*JAG1* overexpression constructs were obtained through gateway cloning. Donor plasmid (Human *JAG1* in pDONR223, clone no. HsCD00353838) was obtained from DNASU Plasmid Repository^32^ and cloned into pLenti7.3/V5-DEST plasmid (Invitrogen, V53406). Control *LacZ* expressing vector (pLenti7.3/V5-GW/*LacZ*) was included in the pLenti7.3/V5-DEST Gateway Vector Kit. Lentiviral particles were produced by co-transfection with 2 µg pCMV-VSV-G, 4 µg pCMV-dR8.2-dvpr, and 4 µg of the expression plasmid into 6 × 10^6^ 293T cells (ATCC, CRL-3216) using 20 µl Fugene HD (Promega). Cell culture media of 293T cells were harvested at 24 and 48 h post transfection. After harvesting, lentiviral transduction was performed in the presence of protamine sulfate, then incubating overnight. Selection of vector-expressing, GFP^+^ cells was achieved by flow cytometry sorting and Jagged-1 overexpression was confirmed by western blot.

### Immunofluorescence

Adherent cultures and hydrogels were fixed with 4% paraformaldehyde at room temperature, then permeabilized using 0.1% TritonX-100 and incubated with blocking solution (PBST with 10% goat serum and 3% BSA) for 1 h at room temperature and stained with the appropriate primary antibody in blocking buffer for 1–2 h at room temperature or overnight at 4 °C. The samples were then washed with PBS, incubated with a secondary antibody, washed and stained with DAPI or Hoechst 34580. Primary antibodies used in this study were: DDR1 (5583, Cell Signaling Technology, clone D1G6, 1:100), E-Cadherin (ab1416, Abcam, Clone HECD-1, 1:100), JAG1 (PA5-46970, Invitrogen, 1:100), Notch1 (4380, Cell Signaling Technology, clone D6F11, 1:200), CK18 (4548 Cell Signaling Technology, clone DC10, 1:250), p63 (13109, Cell Signaling Technology, clone D2K8X, 1:250), CK14 (RB-9020-P, Thermo Fisher, 1:200). Secondary antibodies used were: Rat anti-mouse-APC (550874, BD Bioscience, 1:500), Donkey anti-rabbit-AF555 (A-3157, Invitrogen, 1:500), Donkey anti-rabbit-AF555 (A-31572, Invitrogen, 1:500), Donkey anti-goat-AF488 (A-11055, Invitrogen, 1:500). Donkey-anti-mouse-AF555 (A-31570, Invitrogen, 1:500). Dyes and probes used for immunofluorescence: Phalloidin-AF647 (A22287, Life Technologies, 1:400 of 400× stock), DAPI (D1306, Life Technologies, 1 ug/ml), Hoechst 34580 (H21486, Life Technologies, 1 µg/ml).

### Microscopy and image analysis

Immunofluorescence images were captured using a Zeiss LSM 700, Zeiss LSM 710, and Zeiss LSM 800 (Zeiss Microscopy, Thornwood, NY, USA). Brightfield images were captured using a Zeiss Axiophot 25 (Zeiss Microscopy) and Nikon Eclipse Ti-U (Nikon Microscopy), using SPOT 5.6 software.

Analysis of immunofluorescent staining and EdU staining captured by confocal fluorescent microscopy was performed using Zen software, versions Blue 2.6 and Black edition (Zeiss). Analysis of brightfield images, including size measurements, was performed using ImageJ software, version 1.53e^33^.

### Western blotting

To isolate protein from whole-cell lysis for immunoblotting, cells were lysed in RIPA buffer (10 mM Tris, 150 mM NaCl, 1 mM EDTA, 0.1% SDS) supplemented with protease inhibitor cocktail (Roche). Protein samples were separated by SDS-PAGE, transferred onto nitrocellulose membrane and blocked with 5% milk. Membranes were incubated with primary antibody overnight at 4 °C and secondary antibody for 1 h at room temperature. Immunoblot membranes were developed with chemiluminescent substrate (Thermo Fisher Scientific) and imaged using Chemidoc XRS+ with Image Lab 6.0.1 software (BioRad, Hercules, CA). Densitometry quantification was performed using Image J, version 1.53e. Primary antibodies used were: anti-total DDR1 (5583; Cell Signaling Technology, clone D1G6, 1:1000), GAPDH-HRP (3683, Cell Signaling Technology, clone 14C10, 1:1000), anti-cleaved NOTCH1 (4147, Cell Signaling Technology, clone D3B8, 1:1000), anti-phospho-DDR1 Y513 (14531, Cell Signaling Technology, clone E1N8F, 1:1000), anti-JAG1 (2620, Cell Signaling Technology, clone 28H8, 1:1000) and anti-β-Tubulin- HRP (5346; Cell Signaling Technology, clone 9F3, 1:1000). Secondary antibody used was anti-rabbit-HRP (7074, Cell Signaling, 1:5000).

### Flow cytometry

Patient-derived mammary tissues (*n* = 3) were grown in hydrogels for 12 days in the presence or absence of DDR1 inhibitor (2 μM). Cells were isolated from hydrogels using collagenase followed by trypsin, as described above for colony and mammosphere assays. Cells were incubated for 90 min on ice with the following antibodies: EpCAM-PE (347198, BD Biosciences, clone EBA-1, 1:5), EpCAM-APC (347200, BD Biosciences, clone EBA-1, 1:5), CD49f-FITC (555735, BD Biosciences, clone G0H3, 1:5). Subsequent analyses on luminal and basal cell populations were performed using the following antibodies: JAG1-PE (94449, Cell Signaling Technology, clone D4Y1R 1:50) or DDR1-PE (ab253251, Abcam, clone 51D6, 1:20). DDR1+ and Jagged-1+ population gating was determined based on fluorescence minus one (FMO) controls. Flow cytometry was performed on BD LSR II flow cytometer at the Tufts Flow Cytometry Core. Flow cytometry data were collected using FACS Diva, version 6.2. Flow cytometry data analysis was performed on FlowJo software v10.7. All analyses were performed in compliance with MiFlowCyt standards^34^. The complete report is available upon request. A figure exemplifying the gating strategy for flow cytometry experiments in this study is provided in the Supplementary Information (Supplementary Fig. 6).

### Seq-well single-cell RNA sequencing

Libraries were prepared from single cells from dissociated hydrogel-grown tissues that were isolated, co-loaded with beads onto a microwell array and lysed. Bead-bound, barcoded mRNA was reverse transcribed and sequenced. The complete Seq-well protocol was previously described in detail^14^. Sequencing was carried out on an Illumina NextSeq 500 to achieve paired end reads.

### scRNA-seq analysis

Read alignment was performed as previously described^14^. Briefly, reads were aligned to GRCh38, and individual reads from read 2 were tagged by their corresponding read 1 pair using the 12-bp cellular barcode and the 8-bp UMI. Afterward, reads were deconvoluted to individual cells using Drop-seq tools (http://mccarrolllab.com/dropseq). Barcodes and UMIs were collapsed using a Hamming distance of 1 to obtain a digital gene expression matrix. For each cell within the digital gene expression matrix, UMI-collapsed gene expression was normalized by scaling by the total number of transcripts and multiplying by a factor of 10,000. Scaled gene expression was then natural-log transformed.

The Seurat package (v2.2.0) was used to perform clustering analysis^35^. First, cells with less than 500 genes and with mitochondrial content greater than 7.5% were removed from the analysis. Genes detected in less than 3 cells were dropped from the analysis. 7193 highly variable genes, selected using the MeanVarPlot function in Seurat with a low cutoff of 0.0125 and a high cutoff of 5 for dispersion and an average expression cutoff of 0.4, were used to perform principle component analysis. The top 8 principle components were used to perform t-distributed stochastic neighbor embedding (tSNE) analysis. Clusters were called using the FindClusters function with a resolution of 1^35^. Clusters corresponding the indicated clusters were classified using gene set enrichment analysis (GSEA) with the input being a pre-ranked list of gene enrichment in a specific cluster relative to all the remaining cells^36^. *NOTCH1* peaks were defined from the *NOTCH1* dynamics peaks bed file from Wang et al.^22^. Genes associated with peaks were defined by HOMER (v4.9.1), using the annotatePeaks command^37^. Additionally, to calculate *p*-values for individual genes, we applied the likelihood ratio test (LRT). To highlight cell cycle genes, we used genes from Macasko et al.^16^. To identify significantly enriched overlaps, we used the piano package to perform overlap analysis on Bonferroni corrected significant genes. Significant genes were selected from the DDR1i treated cells relative to the cells from the remaining conditions and from the DDR1-inh released cells relative to the remaining conditions. The Monocle v2.6.1 package was used to perform lineage trajectory reconstruction on cells identified from the Seurat analysis^38^. Size factors and dispersion estimates were calculated using the default parameters. Genes used for dimensionality reduction were limited to those that were expressed in at least 10 cells with a mean expression of at least 0.01 and an empirical dispersion of at least 0.4 times the dispersion fit. Dimensionality reduction and pseudotime calculations were performed using the default parameters.

### Statistics

All statistics, excluding those performed for single cell and screen analyses, were performed using GraphPad Prism 7. Two-sided Student’s *t*-tests were performed, unless otherwise specified.

### Reporting summary

Further information on research design is available in the Nature Research Reporting Summary linked to this article.

## Supplementary information

Supplementary Information

Reporting Summary

## Data Availability

scRNA-seq data generated in this study have been deposited deposited in Gene Expression Omnibus, accession number “GSE162296”. All other relevant data supporting the key findings of this study are available within the article and its Supplementary Information files or from the corresponding author upon reasonable request. Source data are provided with this paper.

## Source data

Source data
