# Supplementary material for: Breast tissue regeneration is driven by cell-matrix interactions coordinating multi-lineage stem cell differentiation through DDR1
Source: Nat Commun. 2021 Dec 10;12:7116. doi: 10.1038/s41467-021-27401-6 (PMC8664951; doi:10.1038/s41467-021-27401-6)
Supplement: Supplementary file 1 — Supplementary Information [file 41467_2021_27401_MOESM1_ESM.pdf]

SUPPLEMENTARY INFORMATION

Breast tissue regeneration is driven by cell-matrix interactions coordinating multi-lineage stem cell differentiation through DDR1

Authors: Gat Rauner[1], Dexter X. Jin[2,3], Daniel H. Miller[2,3], Todd M. Gierahn[4], Carman M. Li[5], Ethan S. Sokol[2,3], Yu-Xiong Feng[2], Robert A. Mathis[2,3] , J. Christopher Love[4,6,7], Piyush B. Gupta[1,2,3,8]*, Charlotte Kuperwasser[1,8]*

Affiliations:

[1] Department of Developmental, Chemical & Molecular Biology, Tufts University, Boston, MA 02111, USA

[2] Whitehead Institute for Biomedical Research, Cambridge, MA 02142, USA.

[3] Department of Biology, Massachusetts Institute of Technology, Cambridge, MA 02139, USA.

[4] Koch Institute for Integrative Cancer Research, Massachusetts Institute of Technology, Cambridge, MA 02142, USA.

[5] Department of Cell Biology, Ludwig Center at Harvard, Harvard Medical School, Boston, MA 02115, USA.

[6] Broad Institute of MIT and Harvard, Cambridge, MA 02142, USA.

[7] Ragon Institute of MGH, MIT and Harvard, Cambridge, MA 02129, USA.

[8] Laboratory for the Convergence of Biomedical, Physical, and Engineering Sciences, Tufts University School of Medicine, Boston, MA 02111, USA.

These authors contributed equally: Gat Rauner, Dexter X. Jin, Daniel H. Miller.

*Correspondence to: Piyush B. Gupta (E-mail: piyush.gupta@tufts.edu); Charlotte Kuperwasser (E-mail: charlotte.kuperwasser@tufts.edu)

This PDF file includes:

Supplementary figures 1- 6
Supplementary table 1

Supplementary Fig. 1.

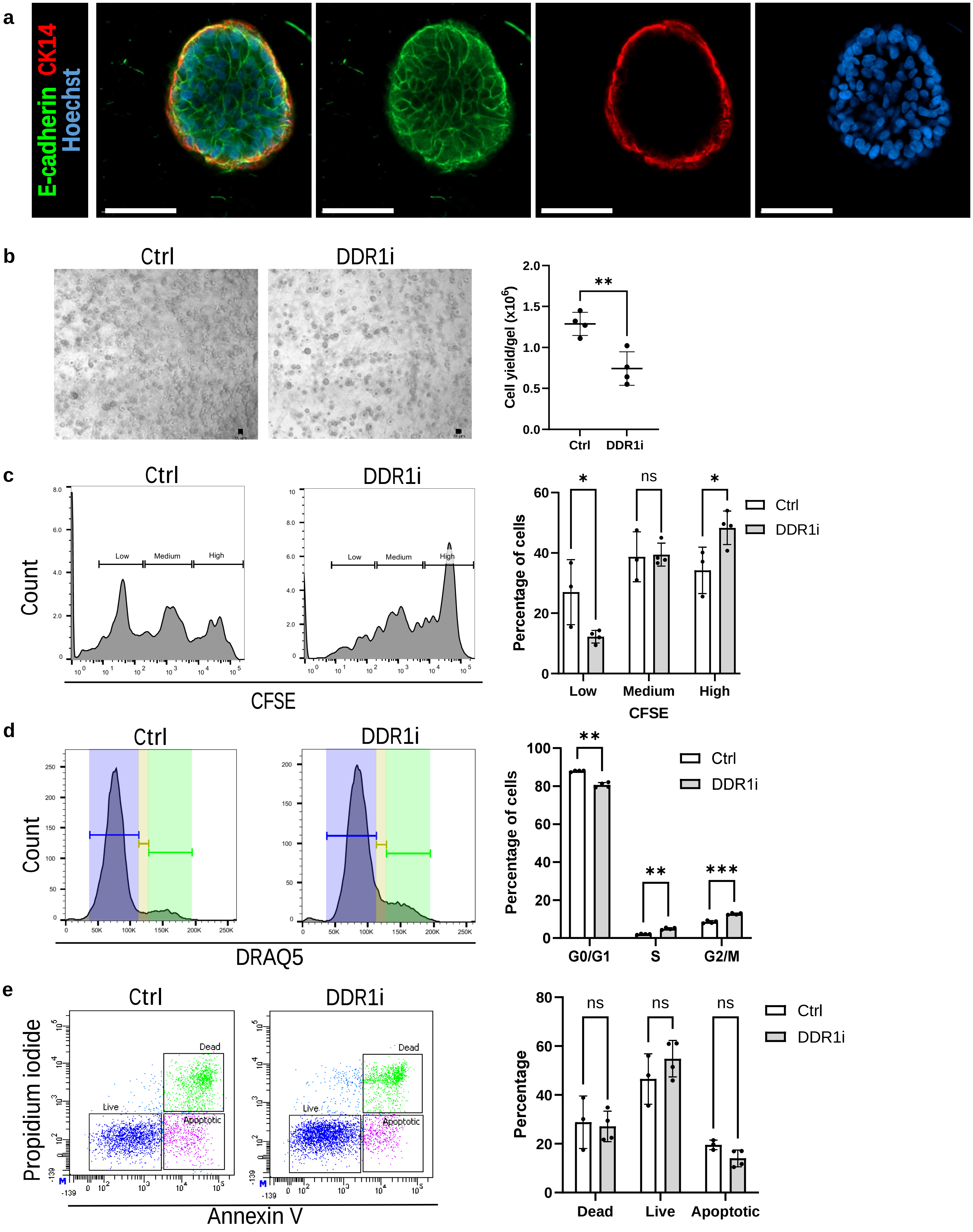

Supplementary Fig. 1. MCF10A cells exhibit bi-lineage differentiation in 3D collagen and DDR1 inhibition does not increase proliferation or apoptosis. **(a)** Immuno-fluorescent staining of organotypic lobular structured that developed from MCF10A cells cultured for 10 days in collagen gel, showing expression and localization of lineage markers: luminal E-cadherin (green) and basal CK14 (red). Scale bar = 50µm. Results are representative of three repeats of this experiment.**(b)** Bright-field images (left) of representative control and DDR1i-treated MCF10A cells grown in collagen gels, and quantification (right) of the average live cell yield from these gels. Data was derived from n=4 gels. P=0.0061 (two-tailed Student's t-test). **(c)** CFSE dye dilution proliferation analysis of MCF10A cells cultured in collagen gels in the presence or absence of DDR1 inhibitor. Statistical analysis compares the percentage of cells with "low" (P=0.028), "medium" (P=0.998) and "high" (P=0.037) CFSE intensity between the Ctrl and DDR1i groups (ordinary one-way ANOVA with Sidak's multiple comparisons test). Data was derived from n=3 and n=4 independent gels for the Ctrl and DDR1i groups, respectively. **(d)** Cell cycle analysis of MCF10A cells cultured in collagen gels in the presence or absence of DDR1 inhibitor. Statistical analysis compares the percentage of cells in G0/G1 (P=0.0026), S (P=0.0012) and G2/M (P=0.0004) between the Ctrl and DDR1i groups (ordinary one-way ANOVA with Sidak's multiple comparisons test). Data was derived from n=4 gels. **(e)** Annexin V apoptosis analysis of MCF10A cells cultured in collagen gels in the presence or absence of DDR1 inhibitor. Data was derived from n=3 and n=4 independent gels for the Ctrl and DDR1i groups, respectively. * indicates $p<0.05$; ** indicates $p<0.01$; *** indicates $p<0.001$. All graphs in this figure are presented as mean values +/- SD. Source data are provided as a Source Data file.

Supplementary Fig. 2.

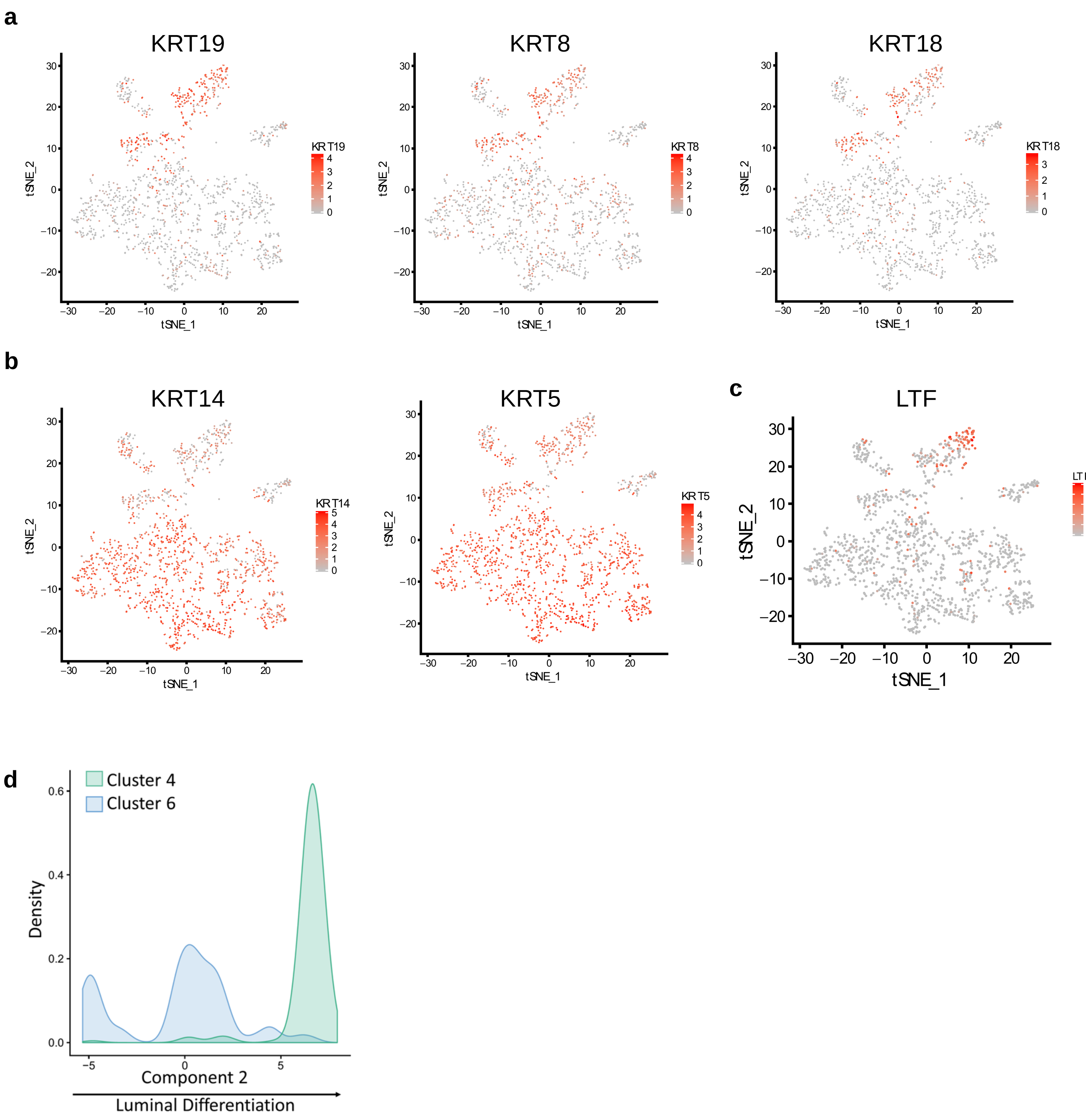

Supplementary Fig. 2. Additional lineage analyses of scRNA-seq. (a) Single cell expression of luminal cytokeratins overlaid as a heatmap on the tSNE plot. **(b)** Single cell expression of basal cytokeratins overlaid as a heatmap on the tSNE plot. **(c)** Single cell expression of LTF overlaid as a heatmap on the tSNE plot. **(d)** Kernel density plots depicting the distribution of cells from clusters 4 and 6 along the luminal differentiation axis.

Supplementary Fig. 3.

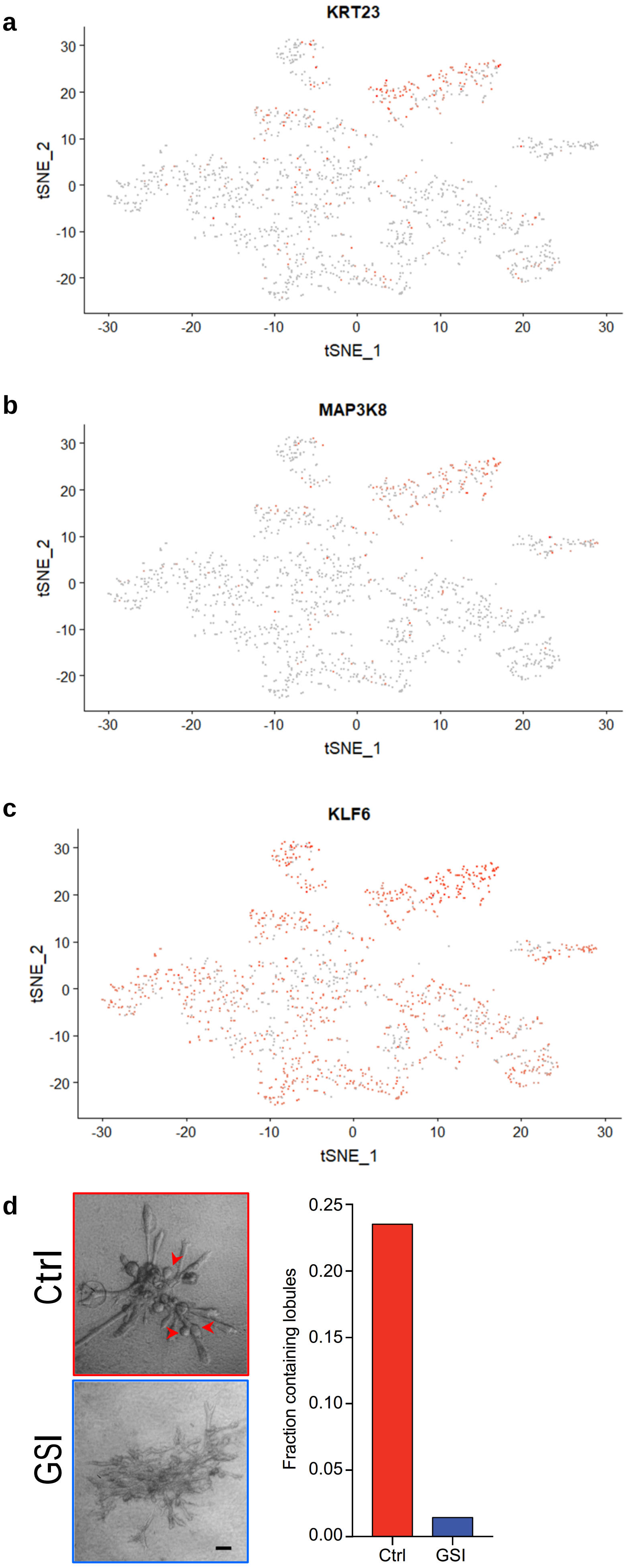

Supplementary Fig. 3. Lobular development in GSI-treated of human breast tissue. (a-c) Single cell expression of Notch1 target genes overlaid as a heatmap on the tSNE plot. **(d)** Bright-field images (left) of representative control and GSI-treated patient-derived tissues grown in hydrogels for 12 days, and quantification (right) of the fraction of tissues containing lobules. Red arrowheads indicate lobules. Scale bars = 100μm. Source data are provided as a Source Data file.

Supplementary Fig. 4.

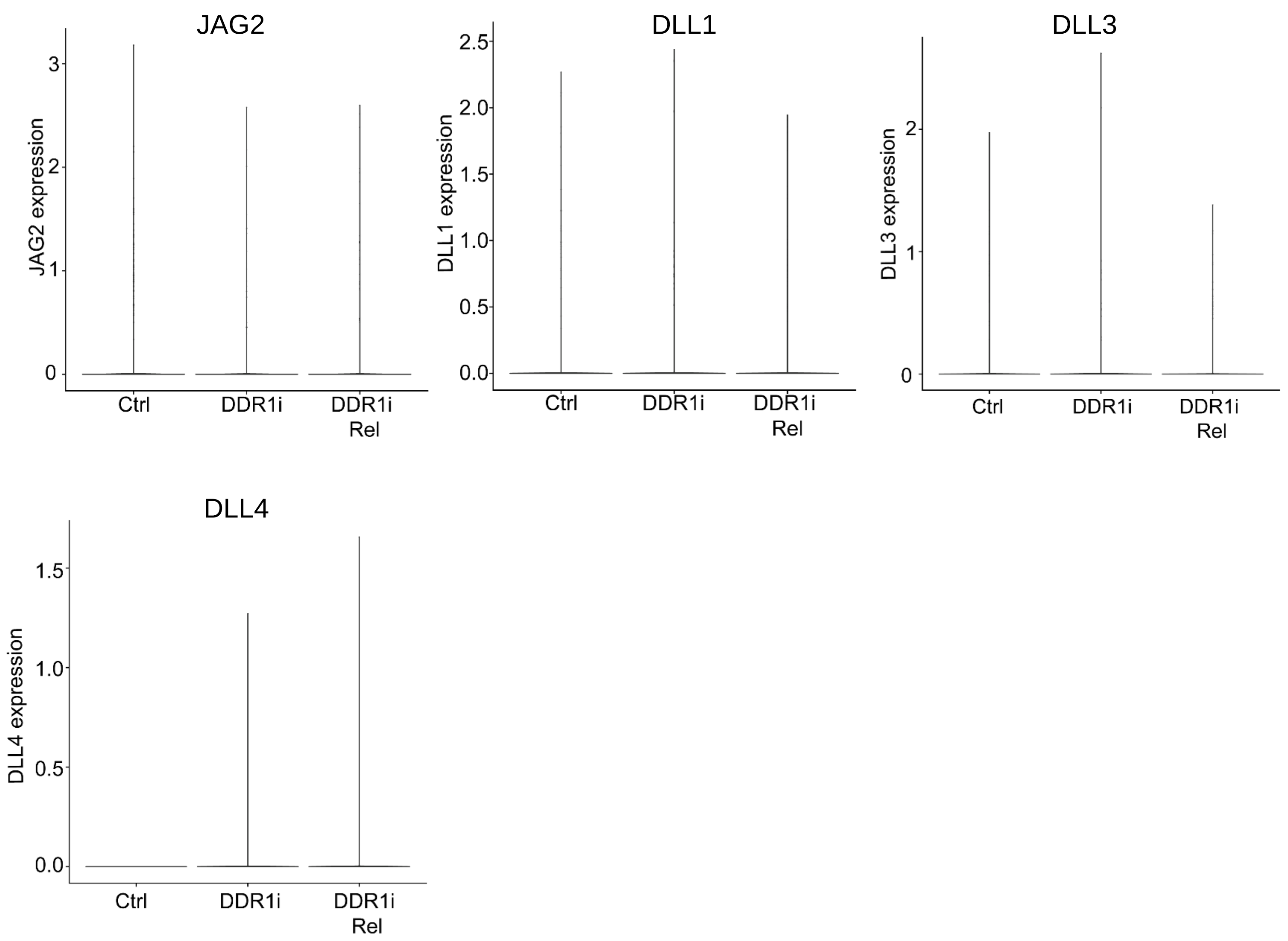

Supplementary Fig. 4. Changes in Notch1 ligand expression upon DDR1 inhibition and release from inhibition. Violin plots depicting expression of Notch1 ligands JAG2, DLL1, DLL3 and DLL4 (DLL2 was not detected) in cells from control, DDR1-inhibited and DDR1-inhibitor released tissues.

Supplementary Fig. 5.

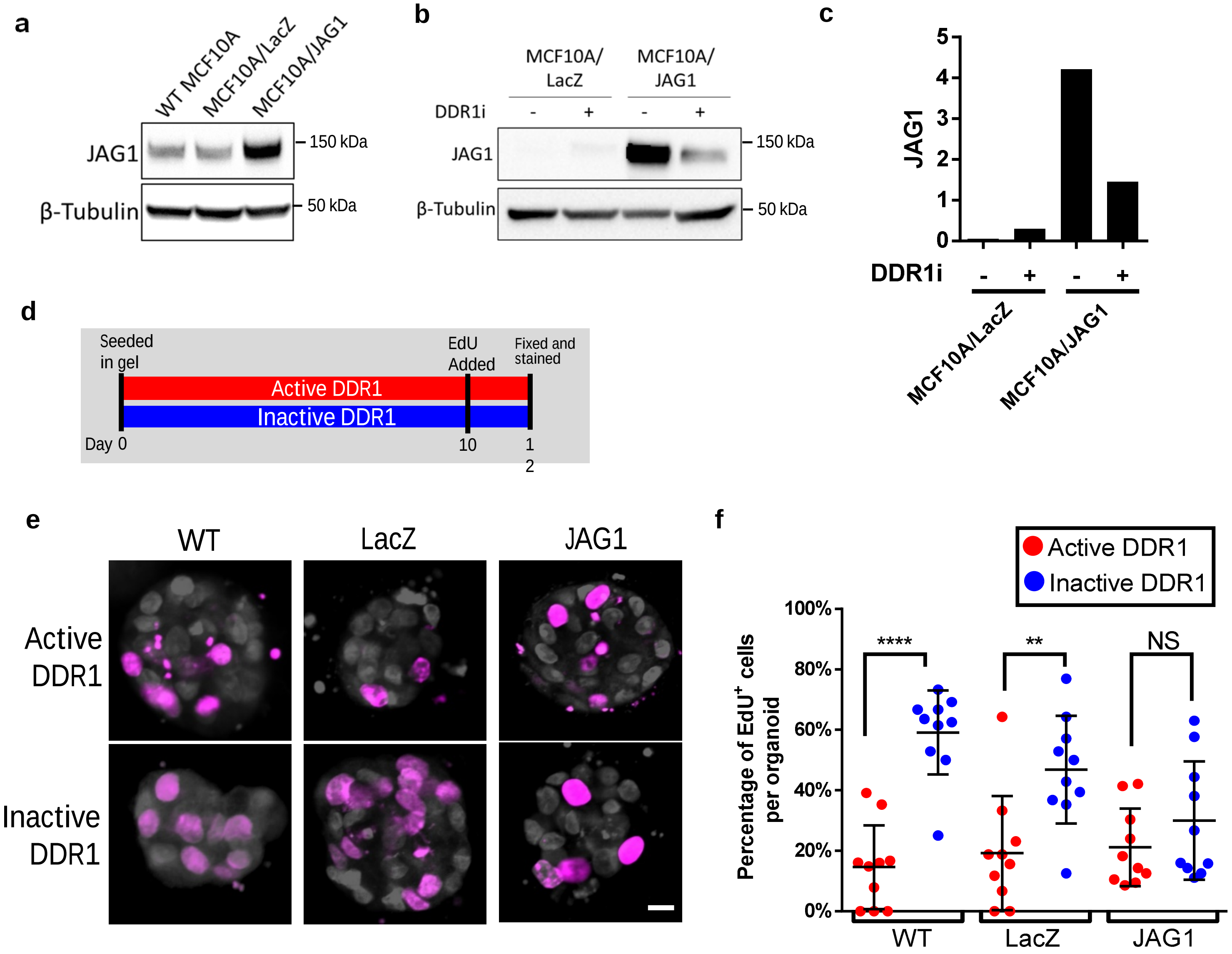

Supplementary Fig. 5. JAG1 overexpression prevents increased DNA replication induced by DDR1i. **(a)** Western blot for Jagged-1 in 2D-cultured parental cells (WT) and cells transduced with construct for JAG1 or LacZ overexpression. Blot is representative of two independent experiments. **(b)** Western blot for Jagged-1 in 3D-cultured cells transduced with construct for JAG1 or LacZ overexpression, in the presence or absence of DDR1 inhibitor. Blot is representative of two experiments. **(c)** Densitometric quantification of blot shown in (b). **(d)** Timeline of experiment: cells were cultured in 3D collagen gels with or without DDR1i. EdU was introduced on day 10. Gels were fixed for analysis on day 12. **(e)** Confocal microscope imaging of EdU+ cells (violet). Nuclei were stained with Hoechst (gray). Scale bar = 10μm. This experiment was performed once. **(f)** Analysis of the percentage of EdU+ cells per organoid in WT, LacZ or JAG1 overexpressing MCF10A cells, cultured with or without DDR1i. Data was derived from n=10 organoids per treatment group in each of the cell lines. $P=4*10^{-7}$, 0.001 and 0.549 comparing Active and Inactive DDR1 groups within WT, LacZ and JAG1 cell lines, respectively (ordinary two-way ANOVA with Sidak's multiple comparisons test). Data are presented as mean values +/- SD. **** indicates $p<0.0001$, ** indicates $p<0.01$, NS indicates no statistically significant difference. Source data are provided as a Source Data file.

Supplementary Fig. 6.

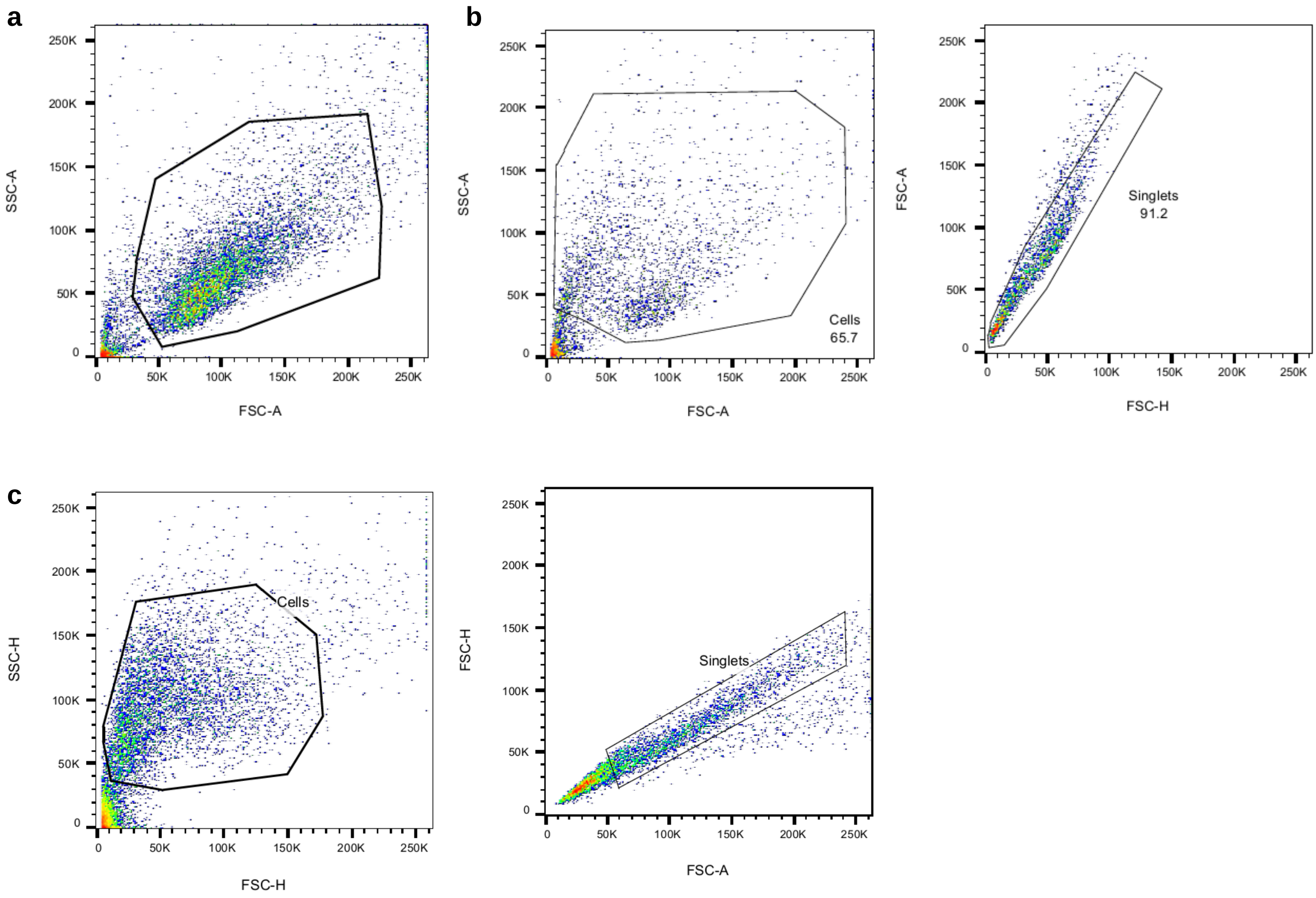

Supplementary Fig. 6. Gating strategy for flow cytometry experiments. (a) Representative gating strategy to select for cells, based on SSC-A and FSC-A parameters, prior to flow cytometry analysis of lineage distribution changes upon DDR1 inhibition and release from inhibition, presented in Fig. 2j. **(b)** Representative gating strategy to select for cells, based on SSC-A and FSC-A parameters, followed by selection for singlets, based on FSC-A/FSC-H. This gating strategy was used prior to flow cytometry analysis of DDR1 expression in luminal and basal cells (Fig. 4c), and of JAG1 expression changes upon DDR1 inhibition (Fig. 5d). **(c)** Representative gating strategy to select for cells, based on SSC-H and FSC-H parameters, followed by selection for singlets, based on FSC-H/FSC-A. This gating strategy was used prior to flow cytometry for CFSE proliferation analysis, cell cycle analysis, and Annexin V apoptosis analysis of MCF10A cells (Supplementary Fig. 1 b,c,d).

Supplementary Table 1.

	FC	-log10(p-Val)
DDR1	2.272818	3.154902
ADCK1	0.9916951	2.221849
EIF2AK1	0.9824635	1.958607
MAP3K7	1.346587	1.928118
TESK2	1.317204	1.920819
PRKAA2	0.9593607	1.832683
MAPK7	0.6535482	1.739929
STK36	0.7493176	1.737549
FYN	1.041067	1.696804
CDK17	1.054925	1.643974
GRK1	1.021555	1.493495
BMP2K	0.719038	1.429457
ERN1	0.6387768	1.336299
TYRO3	-0.427167	2.102373
HSPB8	0.3589076	1.906578
TEK	0.4594089	1.649752
AKT1	0.3429806	1.5867
MAPKAPK3	0.1961916	1.585027
STK40	0.4335426	1.575118
MAPK10	0.2457881	1.5157
HCK	0.5690671	1.501689
ADCK4	0.1101253	1.425969
BUB1	0.4954995	1.417937
CDC42BPG	0.5588799	1.39794
MST1R	0.1176791	1.396856
SIK3	0.3988651	1.340084
EPHA2	0.5252816	1.335358
ZAK	0.2777371	1.330683
CAMKV	-0.1284809	1.327902
RIPK1	0.4661416	1.313364
CAMK1G	0.4534567	1.307153
NLK	-0.0707567	1.306273

Supplementary Table 1. Kinase sgRNAs enriched in secondary organoids following CRIPSR-Cas9 screen. sgRNAs directed at 32 kinases were enriched in the secondary organoids based on a -log10(p-value) of 1.3. RIGER-E was used to calculate an FDR corrected significance, p-values are adjusted for multiple tests.
