# Supplementary material for: Breast tissue regeneration is driven by cell-matrix interactions coordinating multi-lineage stem cell differentiation through DDR1
Source: Nat Commun. 2021 Dec 10;12:7116. doi: 10.1038/s41467-021-27401-6 (PMC8664951; doi:10.1038/s41467-021-27401-6)
Supplement: Supplementary file 2 — Reporting Summary [file 41467_2021_27401_MOESM2_ESM.pdf]

nature portfolio

Corresponding author(s): Charlotte Kuperwasser

Last updated by author(s): Oct 29, 2021

Reporting Summary

Nature Portfolio wishes to improve the reproducibility of the work that we publish. This form provides structure for consistency and transparency in reporting. For further information on Nature Portfolio policies, see our Editorial Policies and the Editorial Policy Checklist.

Statistics

For all statistical analyses, confirm that the following items are present in the figure legend, table legend, main text, or Methods section.

n/a	Confirmed	
☐	☒	The exact sample size (n) for each experimental group/condition, given as a discrete number and unit of measurement
☐	☒	A statement on whether measurements were taken from distinct samples or whether the same sample was measured repeatedly
☐	☒	The statistical test(s) used AND whether they are one- or two-sided<br>*Only common tests should be described solely by name; describe more complex techniques in the Methods section.*
☐	☒	A description of all covariates tested
☐	☒	A description of any assumptions or corrections, such as tests of normality and adjustment for multiple comparisons
☐	☒	A full description of the statistical parameters including central tendency (e.g. means) or other basic estimates (e.g. regression coefficient) AND variation (e.g. standard deviation) or associated estimates of uncertainty (e.g. confidence intervals)
☐	☒	For null hypothesis testing, the test statistic (e.g. F, t, r) with confidence intervals, effect sizes, degrees of freedom and P value noted<br>*Give P values as exact values whenever suitable.*
☒	☐	For Bayesian analysis, information on the choice of priors and Markov chain Monte Carlo settings
☒	☐	For hierarchical and complex designs, identification of the appropriate level for tests and full reporting of outcomes
☒	☐	Estimates of effect sizes (e.g. Cohen's d, Pearson's r), indicating how they were calculated

Our web collection on statistics for biologists contains articles on many of the points above.

Software and code

Policy information about availability of computer code

Data collection	Bright field microscopy images were collected using SPOT 5.6 software.<br>Confocal microscopy images were collected using Zen software, versions Blue 2.6 and Black edition.<br>Flow cytometry data was collected using FACS Diva, version 6.2.<br>Western blot membranes were imaged using Chemidoc XRS+ with Image Lab 6.0.1 software.
Data analysis	Flow cytometry data was analyzed using FlowJo software, version 10.7.<br>Analysis of brightfield images, including size measurements, was done using ImageJ software, version 1.53e.<br>Crispr screen: Data was analyzed using RIGER-E.<br>scRNAseq: Clustering analysis was done using the Seurat package, version v2.2.0.<br>GSEA analysis: Genes associated with peaks were defined by HOMER, version v4.9.1, using the Annotate Peaks command.<br>Lineage trajectory reconstruction: the Monocle package, version 2.6.1, was used, using the default settings.<br>Statistics: all statistics, excluding those performed for single cell and screen analyses, were performed using GraphPad Prism 7.<br>Western blot densitometry was performed using ImageJ software, version 1.53e.

For manuscripts utilizing custom algorithms or software that are central to the research but not yet described in published literature, software must be made available to editors and reviewers. We strongly encourage code deposition in a community repository (e.g. GitHub). See the Nature Portfolio guidelines for submitting code & software for further information.

Data

Policy information about availability of data

All manuscripts must include a data availability statement. This statement should provide the following information, where applicable:

- Accession codes, unique identifiers, or web links for publicly available datasets
- A description of any restrictions on data availability
- For clinical datasets or third party data, please ensure that the statement adheres to our policy

scRNAseq data were deposited in Gene Expression Omnibus (GEO), accession number GSE162296.

Field-specific reporting

Please select the one below that is the best fit for your research. If you are not sure, read the appropriate sections before making your selection.

☒ Life sciences ☐ Behavioural & social sciences ☐ Ecological, evolutionary & environmental sciences

For a reference copy of the document with all sections, see nature.com/documents/nr-reporting-summary-flat.pdf

Life sciences study design

All studies must disclose on these points even when the disclosure is negative.

Sample size	n=3 samples were used. Sample size was decided based on our own experience and on previously published relevant work (Sokol et al., 2016).
Data exclusions	No data was excluded in the this study.
Replication	Experiments were replicated at least 3 times, for 3 biological replicates. All attempts of replication were successful.
Randomization	The same individual samples were used for each treatment group, so multiple measurements of the same sample were performed, with no need for randomizing.
Blinding	Analyses were not blinded, because the same researcher performed the experiment and analyzed the data. The collected measurements were quantifiable in nature, minimizing the chance of bias.

Reporting for specific materials, systems and methods

We require information from authors about some types of materials, experimental systems and methods used in many studies. Here, indicate whether each material, system or method listed is relevant to your study. If you are not sure if a list item applies to your research, read the appropriate section before selecting a response.

Materials & experimental systems

n/a	Involved in the study	
☐	☒	Antibodies
☐	☒	Eukaryotic cell lines
☒	☐	Palaeontology and archaeology
☒	☐	Animals and other organisms
☐	☒	Human research participants
☒	☐	Clinical data
☒	☐	Dual use research of concern

Methods

n/a	Involved in the study	
☒	☐	ChIP-seq
☐	☒	Flow cytometry
☒	☐	MRI-based neuroimaging

Antibodies

Antibodies used

Flow cytometry
EpCAM-PE, BD Bioscience, Cat# 347198, clone, EBA-1, concentration 1:5
CD49f-FITC, BD Bioscience, Cat# 555735, clone GoH3, concentration 1:5
EpCAM-APC, BD Bioscience, Cat# 347200, clone EBA-1, concentration 1:5
DDR1-PE, Abcam, cat# ab253251, clone 51D6, concentration 1:20
JAG1-PE, Cell Signaling Technologies, cat# 94449, clone D4Y1R, concentration 1:50
Immunofluorescence
DDR1 unconjugated, Cell Signaling Technologies, cat# 5583, clone D1G6, concentration: 1:100
E-Cadherin unconjugated, Abcam, cat# ab1416, clone HECD-1, concentration: 1:100
JAG1, Invitrogen, cat# PA5-46970, clone: Polyclonal, concentration: 1:100

Notch1, Cell Signaling Technologies, cat# 4380, clone: D6F11, concentration: 1:200
CK18, Cell Signaling Technologies, cat# 4548, clone: DC10, concentration: 1:250
p63, Cell Signaling Technologies, cat# 13109, clone D2K8X, concentration: 1:250
CK14, Thermo Fisher, cat# RB-9020-P, clone: Polyclonal, concentration: 1:200
Rat anti-mouse-APC, BD Bioscience, cat# 550874, clone: N/A, concentration: 1:500
Donkey anti-rabbit-AF555, Invitrogen, cat# A-3157, clone: N/A, concentration: 1:500
Donkey anti-goat-AF488, Invitrogen, cat# A-11055, clone: N/A, concentration: 1:500
Donkey-anti-mouse-AF555, Invitrogen, cat# A-31570, clone: N/A, concentration: 1:500
donkey anti-rabbit-AF555, Invitrogen, cat# A-31572, clone: N/A, concentration: 1:500
Phalliodin-AF647, Life Technologies, cat# A22287, clone: N/A, concentration: 1:400 of 400x stock solution
DAPI, Life Technologies, cat# D1306, clone: N/A, concentration: 1:1000
Hoechst 34580, Sigma, cat# H21486, clone: N/A, concentration: 1:1000
Western blot
Anti-total-DDR1, Cell Signaling Technologies, cat# 5583, clone: D1G6, concentration: 1:1000
GAPDH-HRP, Cell Signaling Technologies , cat# 3683, clone: 14C10, concentration: 1:1000
Anti-cleaved Notch1, Cell Signaling Technologies , cat# 4147, clone: D3B8 , concentration: 1:1000
p-DDR1 Y513, Cell Signaling Technologies, cat# 14531, clone: E1N8F, concentration: 1:1000
Beta Tubulin-HRP, Cell Signaling Technologies, cat# 5346, clone: 9F3, concentration: 1:1000
JAG1, Cell Signaling Technologies, cat# 2620, clone: 28H8, concentration: 1:1000
Anti-rabbit-HRP, Cell Signaling Technologies, cat# 7074, clone: N/A, concentration: 1:5000

Validation	Validation for species<br><br>EpCAM-PE, BD Bioscience 347198, Supplier verified reactivity with human.<br>CD49f-FITC, BD Bioscience 555735, Supplier verified reactivity with human.<br>EpCAM-APC, BD Bioscience 347200, Supplier verified reactivity with human.<br>DDR1-PE, Abcam ab253251, verified by supplier to react with human cell lines.<br>JAG1-PE, Cell Signaling Technologies 94449, Verified by supplier to react with human cell lines.<br>DDR1 unconjugated, Cell Signaling Technologies 5583, Demonstrated by supplier to react with human cell lines.<br>E-Cadherin unconjugated, Abcam ab1416, verified by supplier to react with human carcinoma cells.<br>JAG1, Invitrogen PA5-46970, Verified by supplier to react with human cells, also verified target by neutralization.<br>Notch1, Cell Signaling Technologies 4380, Demonstrated by supplier to react with human MCF7 cells.<br>CK18, Cell Signaling Technologies 4548, Demonstrated by supplier to react with human cell lines.<br>p63, Cell Signaling Technologies 13109, Demonstrated by supplier to react with human cell lines.<br>CK14, Thermo Fisher RB-9020-P, guaranteed by supplier to react with human.<br>Anti-total-DDR1, Cell Signaling Technologies 5583, Demonstrated by manufacturer to react with the human cell line MCF7.<br>GAPDH-HRP, Cell Signaling Technologies 3683, Demonstrated by manufacturer to react with a human cell line.<br>Anti-cleaved Notch1, Cell Signaling Technologies 4147, Demonstrated by manufacturer to react with the human cell line MCF7.<br>p-DDR1 Y513, Cell Signaling Technologies 14531, Demonstrated by manufacturer to react with a human cell line.<br>Beta Tubulin-HRP, Cell Signaling Technologies 5346, Demonstrated by manufacturer to react with human cell lines.<br>JAG1, Cell Signaling Technologies 2620, Demonstrated by manufacturer to react with a human cell line.<br><br>Validation for application<br><br>Flow cytometry<br>EpCAM-PE, BD Bioscience 347198, Routinely verified for flow cytometry application.<br>CD49f-FITC, BD Bioscience 555735, Routinely verified for flow cytometry application.<br>EpCAM-APC, BD Bioscience 347200, Routinely verified for flow cytometry application.<br>DDR1-PE, Abcam ab253251, demonstrated by supplier for use in flow cytometry application<br>JAG1-PE, Cell Signaling Technologies 94449, demonstrated by supplier for use in flow cytometry application.<br>Immunofluorescence<br>DDR1 unconjugated, Cell Signaling Technologies 5583, Demonstrated by supplier to work for immunofluorescent applications.<br>E-Cadherin unconjugated, Abcam ab1416, verified by supplier to work for immunofluorescence.<br>JAG1, Invitrogen PA5-46970, Demonstrated by supplier to work for immunofluorescent applications.<br>Notch1, Cell Signaling Technologies 4380, Demonstrated by supplier to work for immunofluorescent applications.<br>CK18, Cell Signaling Technologies 4548, Demonstrated by supplier to work for immunofluorescent applications.<br>p63, Cell Signaling Technologies 13109, Demonstrated by supplier to work for immunofluorescent applications.<br>CK14, Thermo Fisher RB-9020-P, Supplier demonstrated for immunohistochemistry.<br>Western blot<br>Anti-total-DDR1, Cell Signaling Technologies 5583, Demonstrated by supplier for WB application.<br>GAPDH-HRP, Cell Signaling Technologies 3683, Demonstrated by supplier to work for WB application.<br>Anti-cleaved Notch1, Cell Signaling Technologies 4147, Demonstrated by supplier to react with human MCF7 cell line for WB application.<br>p-DDR1 Y513, Cell Signaling Technologies 14531, Demonstrated by supplier to work for WB application.<br>Beta Tubulin-HRP, Cell Signaling Technologies 5346, Demonstrated by supplier to work for WB application.<br>JAG1, Cell Signaling Technologies 2620, Demonstrated by supplier to work for WB application.

Eukaryotic cell lines

Policy information about cell lines

Cell line source(s)	MCF10A from ATCC
Authentication	Cell line was authenticated by Genetica/LabCorp, using STR profiling
Mycoplasma contamination	Cell line tested negative for mycoplasma
Commonly misidentified lines<br>(See ICLAC register)	None used in this study.

Human research participants

Policy information about studies involving human research participants

Population characteristics	Females with no clinical pathology of the breast, undergoing breast reduction mammoplasty.
Recruitment	Recruitment of tissue donors was done via the Tufts Medical Center Biorepository, and informed consent was obtained from all human research participants. All tissue samples were de-identified.
Ethics oversight	In accordance with 45 CFR 46.104(d)(4) the Tufts Medical Center/Tufts University Health Sciences IRB determined that the study is exempt. IRB# 13521

Note that full information on the approval of the study protocol must also be provided in the manuscript.

Flow Cytometry

Plots

Confirm that:

- [x] The axis labels state the marker and fluorochrome used (e.g. CD4-FITC).
- [x] The axis scales are clearly visible. Include numbers along axes only for bottom left plot of group (a 'group' is an analysis of identical markers).
- [x] All plots are contour plots with outliers or pseudocolor plots.
- [x] A numerical value for number of cells or percentage (with statistics) is provided.

Methodology

Sample preparation	Cells were harvested from hydrogels by collagenase digestion, washed, centrifuged, resuspended in 100ul serum-free medium, and placed in 1.5mL Eppendorf tubes for staining. Cell concentration while staining was 1-5*10^7/mL. Cells were stained with antibodies at a dilution of 1:5. Cells were incubated with the antibodies on ice for 90 minutes, then washed 3 times. Washing consisted of centrifugation (300xg for 5 minutes) and resuspension in fresh serum-free media. Finally, cells were resuspended in PBS+2% horse serum for flow cytometric analysis.
Instrument	BD LSRII SORP (Special Order Research Product)
Software	BD FACSDIVA software
Cell population abundance	Selection of vector expressing GFP+ cells was achieved by flow cytometry sorting. The abundance of GFP+ cells was 32.7 for LacZ controls and 13.5% for cells infected with JAG1 overexpression vector. JAG1 overexpression was confirmed by western blot.
Gating strategy	Events were gated by FSC-A/FSC-H to exclude doublets followed by gating based on FSC/SSC to select for cells. Unstained controls and FMO controls were used to identify positive from negative staining of the relevant antibodies. The gating strategy of the relevant cell population is depicted in the manuscript figures.

- [x] Tick this box to confirm that a figure exemplifying the gating strategy is provided in the Supplementary Information.
